# Human Immunodeficiency Virus Type-1 Myeloid Derived Suppressor Cells Inhibit Cytomegalovirus Inflammation through Interleukin-27 and B7-H4

**DOI:** 10.1038/srep44485

**Published:** 2017-03-24

**Authors:** Ankita Garg, Rodney Trout, Stephen A. Spector

**Affiliations:** 1 Department of Pediatrics, Division of Infectious Diseases, University of California San Diego, La Jolla, California 92093-0672, USA; 2 Rady Children’s Hospital, San Diego, California, 92123, USA

## Abstract

HIV/CMV co-infected persons despite prolonged viral suppression often experience persistent immune activation, have an increased frequency of myeloid derived suppressor cells (MDSC) and are at increased risk for cardiovascular disease. We examined how HIV MDSC control CD4^+^ T cell IFNγ response to a CMVpp65 peptide pool (CMVpp65). We show that HIV/CMV co-infected persons with virologic suppression and recovered CD4^+^ T cells compared to HIV(−)/CMV(+) controls exhibit an increase in CD4^+^CX3CR1^+^IFNγ^+^ cells in response to CMVpp65; MDSC depletion further augmented CD4^+^CX3CR1^+^IFNγ^+^ cells and IFNγ production. IL-2 and IFNγ in response to CMVpp65 were enhanced with depletion of MDSC expanded in presence of HIV (HIV MDSC), but decreased with culture of HIV MDSC with autologous PBMCs. CMVpp65 specific CD4^+^CX3CR1^+^IFNγ^+^ cells were also decreased in presence of HIV MDSC. HIV MDSC overexpressed B7-H4 and silencing B7-H4 increased the production of IL-2 and IFNγ from autologous cells; a process mediated through increased phosphorylated (p)-Akt upon stimulation with CMVpp65. Additionally, IL-27 regulated the expression of B7-H4 on HIV MDSC, and controlled CMV-specific T cell activity by limiting CMVpp65-IFNγ production and expanding CD4^+^IL-10^+^ regulatory T cells. These findings provide new therapeutic targets to control the chronic immune activation and endothelial cell inflammation observed in HIV-infected persons.

Human immunodeficiency virus type-1 (HIV) infection leads to progressive destruction of the immune system resulting in progressive CD4^+^ T cell destruction, profound immune suppression and high risk for opportunistic infections in untreated patients^1^,^2^,^3^. Cytomegalovirus (CMV) is a common cause of end organ disease (EOD) in AIDS patients with severely low CD4^+^ cells^2^,^3^. Numerous studies have shown that CMV specific cellular immunity mediated by cytokine producing CD4^+^ and cytolytic CD8^+^ T cells are crucial for the control of CMV infection; T_H_1 cytokines- IFNγ and TNFα produced by CMV-CD4^+^ T cells emerge prior to CMV-specific antibodies and cytolytic CD8^+^ T cells^4^,^5^. Delayed emergence or absence of CMV-CD4^+^ T cells results in deficient CMV-CD8^+^ T cell cytotoxic activity with development of EOD^6^. Paradoxically, persistent expression of IFNγ by CMV-specific effector T cells results in chronic inflammation and immune activation contributing to immune senescence and cardiovascular complications^7^,^8^,^9^,^10^,^11^. Thus, CMV-CD4^+^ T cells are regulators of protective immunity and immunopathology.

Recently, we showed that an increased number of CD11b^+^CD33^+^CD14^+^HLA DR^−/lo^ monocytic myeloid-derived suppressor cells (MDSC) is present in HIV-infected individuals with replicating virus when compared to HIV-uninfected controls^12^. Animal studies show that MDSC utilize multiple mechanisms to suppress innate and adaptive immunity; this is dependent on the presence of co-inhibitory and co-stimulatory molecules [Reviewed^13^,^14^,^15^,^16^]. The mechanism(s) by which co-inhibitory receptors present on MDSC potentiate suppressive activity to human infections are not known.

IL-27 is an immune regulatory cytokine belonging to the IL-12 family that is predominantly produced by antigen presenting cells (APC). Initially, IL-27 was thought to be a T_H_1 polarizing cytokine (Reviewed^17^,^18^). Subsequent studies have established that IL-27 and IL-27 signaling exert a suppressive effect on CD4^+^ T cells and limit immune-mediated pathology associated with various pathological conditions^19^,^20^,^21^. HIV-infected individuals when compared to uninfected controls demonstrate low plasma IL-27 levels^22^, down-regulated expression of IL-27R and suppressed production of cytokines in response to IL-27^23^. The effect of IL-27 on the immune response to CMV infection is unknown.

In the present study, we show that HIV/CMV co-infected individuals with undetectable plasma HIV RNA and recovered CD4^+^ T cells as compared to HIV-uninfected CMV(+) controls have increased numbers of CMVpp65-specific IFNγ producing activated CD4^+^CX3CR1^+^ cells and this further increases with the depletion of MDSC. MDSC expanded in the presence of HIV, control CMV specific T cell activation and excess IFNγ production from CD4^+^CX3CR1^+^ cells; this is mediated through IL-27 and the inhibitory ligand B7-H4 expressed on HIV MDSC. We further show that IL-27 regulates IFNγ and IL-10 production from T cells in response to CMV and induces B7-H4 expression on HIV associated MDSC.

## Results

### MDSC regulate CMVpp65 specific CD4^+^CX3CR1^+^IFNγ^+^ T cells in HIV/CMV co-infected individuals

**Figure 1:**
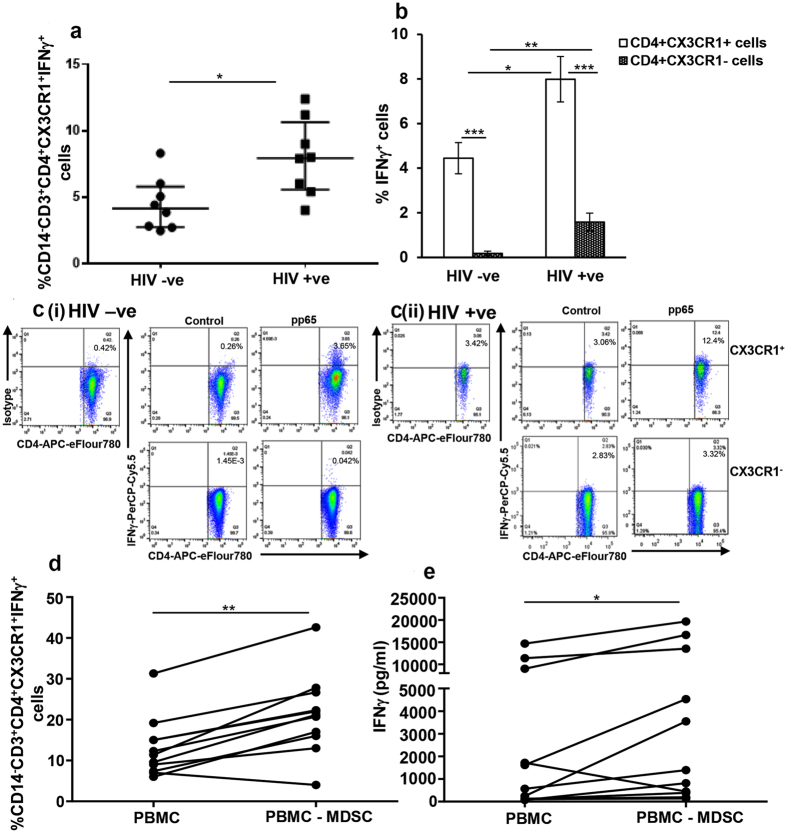
MDSC regulate CD4^+^CX3CR1^+^IFNγ^+^ cells in HIV/CMV co-infected individuals. **(a–c)** All donors were CMV(+) and either HIV-infected (HIV+) or HIV–uninfected (HIV−). PBMCs of HIV(−) and HIV(+) donors were cultured with or without CMVpp65 for 72 hrs. Brefeldin A was added for the last 5 hrs of culture. Cells were surface stained with anti-CD14, -CD3, -CD4 and –CX3CR1, fixed, permeabilized and stained using anti-IFNγ for intracellular IFNγ. (**a**) Percentage of CD14^−^CD3^+^CD4^+^CX3CR1^+^IFNγ^+^ cells was determined by flow cytometry. The plots include observations from 25th to 75th percentile; the horizontal line represents the median value. (**b**) Percentage of IFNγ^+^ cells were determined in CD14^−^CD3^+^CD4^+^CX3CR1^+^ *(open histograms)* and CD14^−^CD3^+^CD4^+^CX3CR1^−^ *(shaded histograms)* subsets. (**c**) Representative dot plots showing CD4^+^IFNγ^+^ cells gated on CD14^−^CD3^+^CD4^+^CX3CR1^+^ cells are shown from HIV-uninfected (ci HIV-ve) and HIV-infected (cii HIV+ve) individual. **(d** and **e)** Freshly isolated PBMCs from CMV(+) HIV-infected individuals on ART and with suppressed viral replication were stained with anti-CD14 and –HLA DR antibodies; CD14^+^HLA DR^−/lo^ MDSC were depleted from PBMCs by flow cytometry. Whole PBMC (PBMC) and MDSC depleted PBMC (PBMC-MDSC) were cultured with or without CMVpp65 for 72 hrs as above. Culture supernatant was stored and cells were stained as detailed above. (**d**) Percentage of CD14^−^CD3^+^CD4^+^CX3CR1^+^IFNγ^+^ cells was determined by flow cytometry. (**e**) Amount of IFNγ in the culture supernatants was determined by ELISA. The histograms in **b** show mean values+/−SD; n = 8 donors. For all other graphs each dot represents an individual donor; *p < 0.05, **p < 0.005, ***p < 0.0005.

In the initial set of experiments, we found that CMVpp65 specific IFNγ responses in HIV-infected individuals on ART with sustained virologic suppression (<50 copies/mL; CD4 T cell >200/mm^3^) were suppressed compared to HIV-uninfected controls (Supplemental Figure S1A and S1B). Previously, we had found that CD4^+^ T cells specific for CMVpp65 produce IFNγ and induce expression of fractalkine (CX3CL1) in endothelial cells^7^,^8^. These results coupled with reports that CX3CR1^+^ T cells are associated with atherosclerosis^10^,^24^ prompted us to examine the frequency of CD4^+^CX3CR1^+^ cells producing IFNγ in response to CMVpp65 by flow cytometry. As expected, the frequency of CD4^+^CX3CR1^+^IFNγ^+^ cells in PBMCs of HIV-uninfected CMV(+) and HIV-infected CMV(+) donors stimulated with CMVpp65 was increased compared to respective unstimulated controls (data not shown). The net CMVpp65 specific frequency of CD4^+^CX3CR1^+^IFNγ^+^ cells in PBMCs of HIV-uninfected CMV(+) donors was significantly less than in HIV-infected CMV(+) donors (4.4 ± 0.7 vs 8.0 ± 1.02; p = 0.02) (Fig. 1a and c- Upper panel). Additionally, CD4^+^CX3CR1^+^ T cells compared to CD4^+^CX3CR1^−^ T cells were the major producers of IFNγ with (8.0 ± 1.02% vs 1.6 ± 0.4%; p = 0.0001) or without (4.4 ± 0.7% vs 0.2 ± 0.1%; p = 0.0005) HIV infection (Fig. 1b and c- Lower panel). For some CMV (+) HIV-uninfected and –infected donors, we cultured PBMC in the presence of CMVpp65 for 48–72 hrs, isolated CD4^+^CX3CR1^−^ and CD4^+^CX3CR1^+^ cells and cultured them on ELISPOT plates coated with anti-IFNγ capture antibodies to determine the frequency of IFNγ producing cells. Consistent with our flow cytometry data, the frequency of CD4^+^CX3CR1^+^ cells producing IFNγ was increased compared to CD4^+^CD3CR1^−^ cells both in HIV-uninfected and –infected groups (Supplemental Figure S1C). Although not reaching statistical significance, the frequency of CD4^+^CX3CR1^+^ cells producing IFNγ trended toward being increased in HIV-infected individuals compared to HIV-uninfected (Supplemental Figure S1C). These data support that a subset of HIV/CMV co-infected individuals with reconstituted CD4^+^ T cell numbers have increased IFNγ producing CD4^+^CX3CR1^+^ cells and may be at increased risk of developing chronic endothelial inflammation and atherosclerosis.

We next hypothesized that MDSC play an important role in regulating the immunologic response to CMV and in controlling excessive IFNγ production. To test this hypothesis, we depleted CD14^+^HLA DR^lo/−^ MDSC from freshly isolated PBMC of CMV(+) HIV uninfected and infected individuals, and stimulated whole PBMC and MDSC depleted PBMC with CMVpp65 for 72 hrs. The quantity of IFNγ in culture supernatant and frequency of CD4^+^CX3CR1^+^IFNγ^+^ cells was determined by ELISA and flow cytometry, respectively. Similar to HIV-uninfected individuals (Supplemental Figure S1D and S1E), the net CMVpp65 specific frequency of CD4^+^CX3CR1^+^IFNγ^+^ was also greater in MDSC depleted PBMC cultures when compared to whole PBMC cultures (21.2 ± 2.9 vs 13 ± 2.3; p = 0.002) (Fig. 1d). The net CMVpp65 IFNγ produced by cells of CMV(+) HIV-infected individuals increased when MDSC were depleted (3,604 ± 1,623 vs 5,570 ± 2,218 pg/ml; p = 0.01) (Fig. 1e). Of note, MDSC depletion from one HIV-infected individual decreased CD4^+^CX3CR1^+^IFNγ^+^ cells and IFNγ production in response to CMVpp65. Nevertheless, the increase in T cell activity was not due to the changes in CD3^+^ T cell percentage or activation since MDSC depleted PBMC when compared to whole PBMC exhibited comparable percentages of CD3^+^ T cells and expression of activation markers (HLA DR, CD69 and CD38) (Supplemental Figure S1F and S1G). These data establish that CD14^+^HLA DR^lo/−^ MDSC regulate the immunologic response to CMV and control excessive IFNγ production in the setting of HIV/CMV co-infection.

### *In vitro* expanded HIV MDSC reproduce the properties of *ex vivo* MDSC

**Figure 2:**
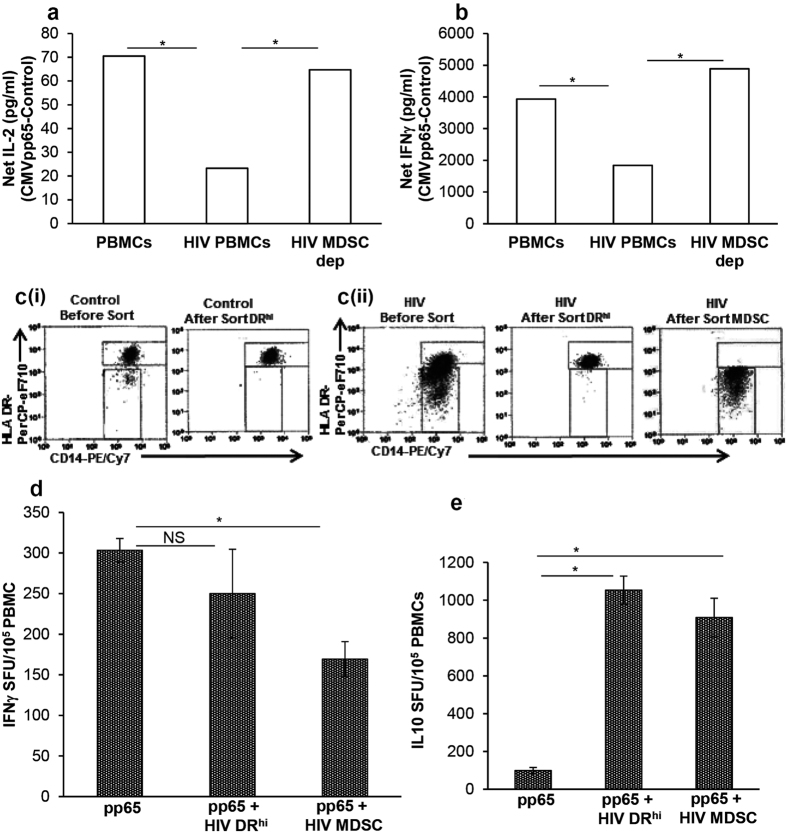
*In vitro* expanded HIV MDSC are functionally similar to MDSC in HIV-infected individuals and modulate CMV specific T cell response. PBMCs of CMV(+) HIV(−) donors were cultured without (PBMCs) or with inactivated HIV_BaL_ (HIV PBMCs) (p24 Ag, 12 ng/10^7^ cells). After 5 days, cells were stained with anti-CD11b, -CD33, CD14, HLA DR. (**a** and **b**) CD14^+^HLA DR^−/lo^ MDSC were depleted from HIV PBMCs. HIV PBMCs, HIV PBMCs and MDSC depleted HIV PBMCs (HIV MDSC dep) were cultured with or without CMVpp65 for 24 and 48 hrs. The amounts of IL-2 (**a**) and IFNγ (**b**) were determined in culture supernatants at 24 and 48 hrs, respectively. Net IL-2 or IFNγ was calculated as CMVpp65 – Control. (**c** and **d**) PBMCs were cultured and stained as above, DR^hi^ and MDSC were sorted. (ci and cii) Representative dot plots show HLA DR vs CD14 in control cells before sort and after sort *(Left panel)* and in HIV_BaL_ treated cells before sort and after sort *(Right panel)*. Cells isolated are >98% pure. (**d** and **e**) Isolated HIV DR^hi^ and MDSC (5 × 10^4^) were cultured overnight with autologous freshly isolated PBMCs (1 × 10^5^) on ELISPOT plates with or without CMVpp65 to determine frequency of (**d**) IFNγ producing cells and (**e**) IL-10 producing cells. Histograms are presented as mean+/−SD; n = 3 donors; *p < 0.05.

In order to understand the mechanism(s) utilized by HIV MDSC to control CMV T cell activation when HIV replication is suppressed, we adopted an *in vitro* approach of MDSC expansion in the presence of non-replicating HIV and studied its effect on CMV-T cell activity. We have previously established that CD14^+^ MDSC expand in the presence of infectious or non-replicating HIV and are able to suppress T cell IFNγ production^12^. In this research, we sought to demonstrate that similar to MDSC present in HIV/CMV co-infected individuals, *in vitro* expanded HIV MDSC modulate CMVpp65 T cell response. For these studies, PBMC of CMV(+) HIV-uninfected donors were cultured with or without heat inactivated HIV and CD14^+^HLA DR^lo/−^ MDSC were depleted as above. Control PBMC, PBMC cultured with HIV and PBMC depleted of HIV MDSC were cultured and stimulated with CMVpp65. Quantities of IL-2 and IFNγ were determined in the culture supernatants after 24 hrs and 48 hrs, respectively. The net CMVpp65 specific IL-2 produced by PBMC cultured without HIV was greater than PBMC cultured with HIV after identical treatment (70.5 ± 15.4 vs 23.3 ± 8.4 pg/ml; p = 0.05) and this increased when HIV MDSC depleted PBMC were cultured with CMVpp65 (23.3 ± 8.4 vs 64.8 ± 9.7 pg/ml; p = 0.04) (Fig. 2a). Likewise, the net CMVpp65 specific IFNγ produced by PBMC cultured without HIV was greater than PBMC cultured with HIV and stimulated identically (3,935.2 ± 1,230 vs 1,839.3 ± 576.1 pg/ml; p = 0.05) and this increased when HIV MDSC depleted PBMC were cultured with CMVpp65 (1,839.3 ± 576.1 vs 4,886.7 ± 1,539 pg/ml; p = 0.05) (Fig. 2b). To further understand the differential role of HIV MDSC and HLA DR^hi^ cells in modulating T cell activation in response to CMVpp65, we cultured PBMCs from HIV-uninfected CMV(+) donors with or without heat-inactivated HIV, and isolated DR^hi^ monocytes and MDSC using flow cytometry. Since very few MDSC expand in control PBMCs, only DR^hi^ monocytes were isolated from this group; isolated cell subsets were >98% pure (Fig. 2c). Each cell subset was cultured with fresh autologous PBMCs with or without CMVpp65 and the frequency of cells producing IFNγ was determined by ELISPOT. The frequency of IFNγ producing cells in PBMCs stimulated with CMVpp65 was higher when compared to PBMCs cultured with isolated MDSC and stimulated identically (303 ± 25 vs 169 ± 37 cells/10^5^ cells; p = 0.03). PBMCs cultured without CMVpp65 failed to produce any IFNγ (data not shown). The frequency of IFNγ producing cells in PBMCs cultured with isolated DR^hi^ cells in presence of CMVpp65 was comparable to PBMCs cultured without DR^hi^ cells (303 ± 25 vs 250 ± 93 cells/10^5^ cells; p = 0.92) (Fig. 2d). In addition, culture of either MDSC or DR^hi^ cells with PBMCs in presence of CMVpp65 increased the frequency of IL-10 producing cells (Fig. 2e). These findings suggest that HIV MDSC contribute to but are not the major modulators of increased CMV IL-10 during CMV/HIV co-infection.

### HIV MDSC regulate CMV specific IFNγ production from CD4^+^CX3CR1^+^ T cells

**Figure 3:**
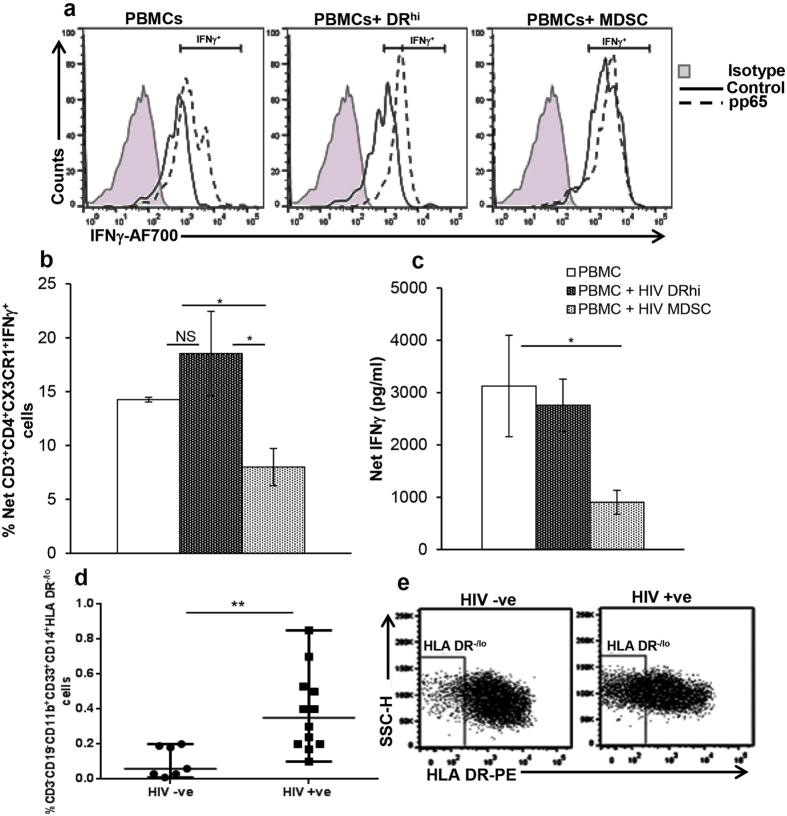
HIV MDSC control CMV specific IFNγ production from CD4^+^CX3CR1^+^ T cells. HIV MDSC were expanded, sorted and cultured with autologous freshly isolated PBMCs as in Fig. 2. Cells were sorted and cultured for 48 hrs, Brefeldin A was added for the last 5 hrs of culture. Cells were surface stained with anti-CD14, -CD3, -CD4 and –CX3CR1, fixed, permeabilized and stained using anti-IFNγ for intracellular IFNγ. **(a)** Representative flow cytometry histograms are shown. **(b)** Percentage of CD14^−^CD3^+^CD4^+^CX3CR1^+^IFNγ^+^ cells was determined by flow cytometry. **(c)** Supernatants were collected and the quantity of IFNγ determined by ELISA. **(d)** Blood obtained from HIV(−) (n = 7) and HIV(+) individuals on ART (HIV+) (n = 12) was stained with anti-CD3,-CD19,-CD11b,-CD33,-CD14, -HLA DR Abs, cells were analyzed as CD3^−^CD19^−^CD11b^+^CD33^+^CD14^+^HLA DR^−/lo^ by flow cytometry. Percentages of MDSCs are shown. **(e)** Representative dot plot with HLA DR^−/lo^ region is shown. For (**d**) Each dot in the plots depict data of each individual donor, the plots include observations from 25^th^ to 75^th^ percentile. The horizontal line represents the median value. For (**b** and **c**), histograms are presented as mean+/−SD; n = 3 donors; *p < 0.05, **p < 0.005.

Having shown that MDSC control IFNγ production by CD4^+^CX3CR1^+^ T cells in response to CMVpp65 (Fig. 2), the next set of experiments were designed to determine the relative contribution of HIV DR^hi^ and MDSC in modulating IFNγ production from CD4^+^CX3CR1^+^ T cells in response to CMVpp65. For these studies, HIV expanded DR^hi^ cells and MDSC from CMV(+) donors were cultured with freshly isolated autologous PBMCs with or without CMVpp65. IFNγ in CD4^+^CX3CR1^+^ cells was determined by flow cytometry. The frequency of CD4^+^CX3CR1^+^IFNγ^+^ T cells in PBMCs stimulated with CMVpp65 was increased compared to PBMCs cultured with HIV MDSC exposed to the same conditions (14.3 ± 0.22% vs 8 ± 1.73%; p = 0.02). No significant difference was observed in CD4^+^CX3CR1^+^IFNγ^+^ T cells when PBMCs were cultured with DR^hi^ cells and stimulated with CMVpp65 (14.3 ± 0.22% vs 18.5 ± 3.9%; p = 0.2]; Fig. 3a and b). We also quantified the amount of IFNγ in the culture supernatants of PBMCs cultured with the different isolated cell subsets. PBMCs stimulated with CMVpp65 produced significantly more IFNγ in the culture supernatants compared to stimulated PBMCs cultured with HIV MDSC (3,127 ± 968 vs 904 ± 228 pg/ml; p = 0.05); no significant difference was observed when PBMCs were cultured with DR^hi^ cells and stimulated with CMVpp65 (3,127 ± 968 vs 2,758 ± 501 pg/ml; p = 0.44; Fig. 3c). This difference was comparable to those observed using ELISPOT (Fig. 2d) and flow cytometry (Fig. 3a and b), and collectively suggest that in the setting of HIV/CMV co-infection, HIV MDSC restrict CMV-specific CD4^+^ T cell IFNγ production. Finally, we determined the frequency of MDSC in the blood of HIV(+) persons receiving ART with sustained undetectable HIV plasma levels and compared them to the frequency of MDSC in HIV(−) controls. Our results show that the frequency of MDSC was significantly higher (0.4 ± 0.2% versus 0.1 ± 0.08%; p = 0.004) in HIV(+) persons compared to HIV(−) controls (Fig. 3d and e). These findings suggest that the level of MDSC in HIV-infected individuals with sustained virologic suppression are higher than HIV-uninfected persons and may regulate immune activation.

### HIV MDSC overexpress inhibitory ligand B7-H4 and regulate CMV-CD4^+^ T cell activity

**Figure 4:**
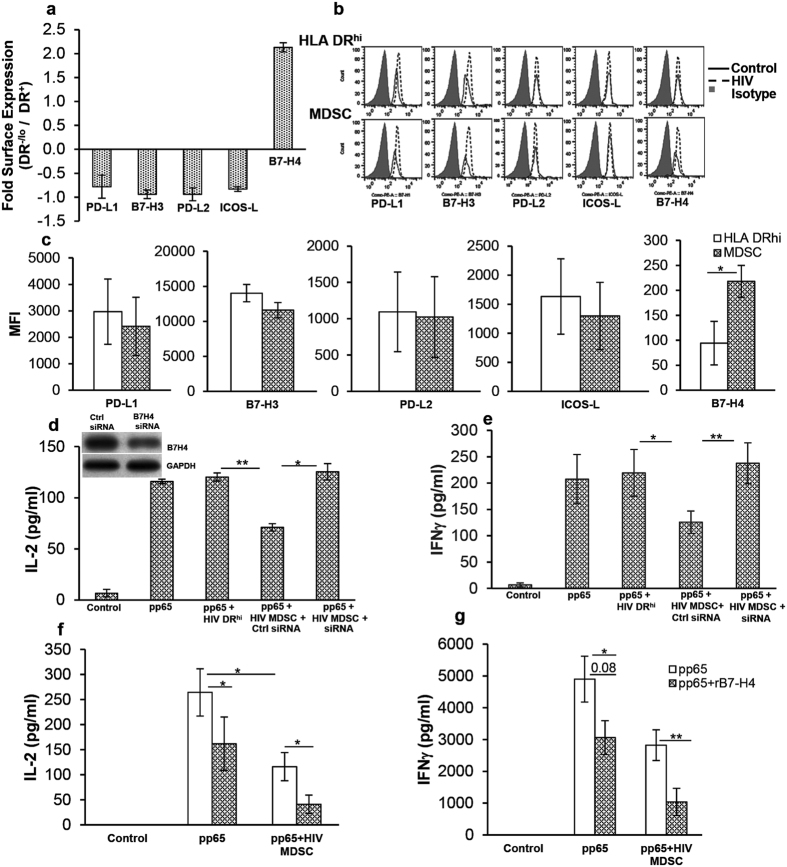
HIV MDSC overexpress the inhibitory ligand B7-H4 and regulate CMV T cell activation. PBMCs from healthy donors were cultured in the presence or absence of HIV_BaL_. After 5 days, cells were stained using anti-CD11b, -CD33, -CD14, -HLA DR and -B7-H1/-B7-H3/-PD-L2/-ICOS-L or –B7-H4. **(a** and **b**) Expression of B7-H1, B7-H3, PD-L2, ICOS-L or B7-H4 was determined by flow cytometry and fold expression of each ligand calculated: MDSC (Control cells - HIV_BaL_ stimulated cells)/DR^hi^ (Control cell - HIV_BaL_ stimulated cells). **(a)** Mean values ± SD from 5 donors are shown **(b)** Representative flow cytometry histogram plot is shown. **(c)** Net Mean Fluorescence Intensity (MFI) of respective inhibitory ligands in DR^hi^ and MDSC was determined: MFI in Control cells – MFI in HIV_BaL_ stimulated. Mean ± SD from 5 donors are shown. (**d** and **e**) PBMCs of CMV(+) HIV(−) healthy donors were cultured with or without non-infectious HIV_BaL_ (p24, 12 ng/10^7^ cells). After 5 days, cells were stained with anti- CD11b, -CD33, CD14, HLA DR; DR^hi^ and MDSC were sorted. MDSC were transfected with control or B7-H4 siRNA (75 nM) using Lipofectamine RNAiMAX reagent (Invitrogen). HIV DR^hi^ cells were cultured in the presence of Lipofectamine RNAiMAX reagent used for transfecting MDSC. After 48–72 hrs, cell lysates of control and B7-H4 siRNA transfected cells were prepared and immunoblotted using anti-GAPDH and –B7-H4 Ab to determine expression of B7-H4. 2.5 × 10^4^ transfected cells were cultured with 5 × 10^4^ freshly isolated autologous PBMCs in the presence or absence of CMVpp65. **(d)** After 18 hrs, IL-2 and **(e)** after 48 hrs IFNγ was determined in culture supernatants by ELISA; partial knockdown of B7-H4 is shown in d. **(f** and **g)** PBMCs of CMV(+) HIV(−) healthy donors were cultured with or without non-infectious HIV_BaL_ (p24, 12 ng/10^7^ cells) and MDSC isolated as above. 0.05 × 10^6^ MDSC were cultured with 0.1 × 10^6^ autologous PBMCs in presence or absence of CMVpp65 with or without rB7-H4 (10 ng/ml). **(f)** After 18 hrs, IL-2 and **(g)** after 48 hrs IFNγ was determined in culture supernatants by ELISA. Mean+/−SD are shown for 3 healthy donors. *p < 0.05, **p < 0.005.

Myeloid cells express inhibitory ligands on their surface which interact with cognate receptors on T cells to control T cell activation and limit IFNγ mediated inflammation. We have previously shown that HIV MDSC modulate T cell function through cell-cell interactions^12^. In these experiments, we hypothesized that inhibitory ligands expressed on HIV MDSC are involved in controlling CMV specific IFNγ production from CD4^+^ T cells. We first examined the surface expression of PD-L1, B7-H3, PD-L2, ICOS-L and B7-H4 on HIV MDSC and compared the expression with DR^hi^ cells. PBMCs from HIV-uninfected donors were cultured with or without non-infectious HIV and expression of ligands was determined by flow cytometry. We observed a 2-fold increase in the expression of B7-H4 on HIV MDSC compared to DR^hi^ cells (Fig. 4a and b); the mean fluorescence intensity (MFI) of B7-H4 on HIV MDSC was greater than B7-H4 MFI on DR^hi^ cells (218.2 ± 92 vs 126 ± 86, p = 0.03) (Fig. 4c). The expression of other ligands on HIV MDSC did not differ significantly when compared to the expression on DR^hi^ cells (Fig. 4a–c).

Next, we examined if B7-H4 on HIV MDSC plays a regulatory role on IFNγ production in response to CMV. PBMCs from CMV(+) HIV-uninfected donors were cultured with or without non-infectious HIV; DR^hi^ cells and MDSC were isolated by flow cytometry. HIV MDSC were transfected with scrambled siRNA or siRNA for B7-H4 and then cultured with autologous PBMCs in the presence or absence of CMVpp65. PBMCs stimulated with CMVpp65 produced more IL-2 compared to unstimulated controls (116 ± 2 vs 6 ± 4 pg/ml; p = 0.001) or PBMCs cultured with HIV MDSC transfected with control siRNA and stimulated with CMVpp65 (116 ± 2 vs 71 ± 3.5 pg/ml; p = 0.005). However, PBMCs cultured with MDSC transfected with B7-H4 siRNA (resulting in ~50% reduction in B7-H4) and stimulated with CMVpp65 produced more IL-2 compared to PBMCs cultured with scrambled siRNA transfected MDSC (125.4 ± 8 vs 71 ± 3.5 pg/ml; p = 0.01; Fig. 4d). Similarly, PBMCs stimulated with CMVpp65 produced more IFNγ compared to unstimulated controls (219 ± 45 vs 6 ± 3 pg/ml; p = 0.01). Culture of PBMCs with isolated DR^hi^ cells did not significantly alter IFNγ production in response to CMVpp65 (219 ± 45 vs 207 ± 46 pg/ml; p = 0.1). Consistent with our previous findings, PBMCs cultured with HIV MDSC in the presence of CMVpp65 produced less IFNγ compared to PBMCs cultured without HIV MDSC (219 ± 45 vs 126 ± 21 pg/ml; p = 0.02); partial knockdown of B7-H4 in HIV MDSC reversed this effect. PBMCs cultured with B7-H4 partially knocked down MDSC produced comparable quantities of IFNγ in the presence of CMVpp65 when compared to PBMCs cultured without MDSC (238 ± 23 vs 219 ± 45; p = 0.1; Fig. 4e). To further establish that B7-H4 regulates MDSC mediated CMVpp65 T cell activity, PBMCs from CMV(+) HIV-uninfected donors were cultured with or without non-infectious HIV; MDSC were isolated by flow cytometry, co-cultured with autologous PBMC in the presence of CMVpp65 with or without rB7-H4; IL-2 and IFNγ were determined in the culture supernatants by ELISA. As previously observed, PBMC cultures stimulated with CMVpp65 produced IL-2 that was inhibited when stimulated PBMC were cultured with HIV MDSC (264.3 ± 47 vs 116.1 ± 28; p = 0.03). Addition of rB7-H4 to PBMC HIV MDSC co-cultures further inhibited IL-2 production in response to CMVpp65 (116.1 ± 28 vs 41.1 ± 18; p = 0.02; Fig. 4f). Similarly, PBMCs cultured with CMVpp65 produced more IFNγ compared to unstimulated controls (data not shown) and this was inhibited when stimulated PBMCs were cultured with HIV MDSC (4900 ± 718.5 vs 2822.5 ± 482.6; p = 0.02). Addition of rB7-H4 to PBMC HIV MDSC co-cultures further inhibited IFNγ production in response to CMVpp65 (2822.5 ± 482.6 vs 1040 ± 428; p = 0.006; Fig. 4g). These findings suggest that HIV MDSC mediate control of CMV-CD4^+^ T cell activation through a B7-H4 dependent mechanism.

**Figure 5:**
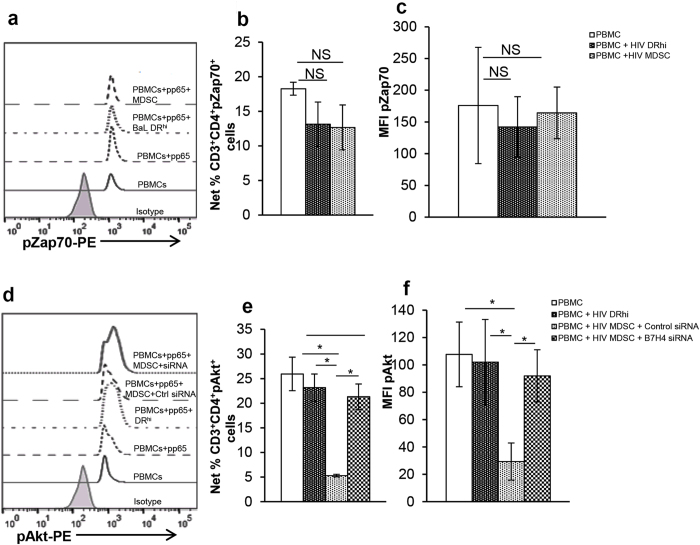
B7-H4 down regulates pAkt to regulate CMV induced T cell activation. To determine the effect of HIV MDSC on CMVpp65 induced pZap70 or pAkt, PBMCs of CMV(+) HIV(−) donors were cultured with or without non-infectious HIV_BaL_ (p24, 12 ng/10^7^ cells). After 5 days, cells were stained with anti -CD11b, -CD33, CD14, HLA DR; DR^hi^ and MDSC were sorted. **(a–c)** Sorted cells and freshly isolated autologous PBMCs were cultured in a ratio of 1:2 (1 sorted cell: 2 PBMCs) with or without CMVpp65 for 15 min. pZap70 was determined as detailed in Methods section. **(a)** Representative flow cytometry histogram plot is shown, **(b)** pZap70 expressed as %Net CD3^+^CD4^+^pZap70^+^ cells and **(c)** MFI of pZap70 in CD3^+^CD4^+^ cells was determined. **(d–f)** Sorted MDSC were transfected with control or B7-H4 siRNA (75 nM) using Lipofectamine RNAiMAX reagent (Invitrogen); HIV DR^hi^ cells were cultured in presence of Lipofectamine RNAiMAX reagent used for transfecting MDSC. After 48–72 hrs, transfected cells and freshly isolated autologous PBMCs were cultured in a ratio of 1:2 (1 sorted cell: 2 PBMCs) with or without CMVpp65 peptide pool for 15–30 min pAkt was determined as above. **(d)** Representative flow cytometry histogram plot is shown, **(e)** pAkt expressed as %Net CD3^+^CD4^+^pAkt^+^ cells and **(f)** MFI of pAkt in CD3^+^CD4^+^ cells was determined. Histograms are presented as mean+/−SD; n = 4 donors. *p < 0.05.

To investigate the mechanism utilized by B7-H4 to control T cell cytokine production in response to CMVpp65, we examined the pZap70, located near the surface membrane of T cells and critical for the signaling cascade and subsequent T cell activation^25^. Our initial experiments were designed to identify the time point at which optimum pZap70 can be detected by flow cytometry. The kinetics of pZap70 expression in CD4^+^ T cells of CMV(+) donors showed the optimum pZap70 at 15 min (Supplemental Figure S2A Upper Panel & S2B) in response to CMVpp65. PBMCs from CMV(+) HIV-uninfected donors were cultured with or without non-infectious HIV. DR^hi^ and MDSC were sorted by flow and cultured with autologous PBMCs with or without CMVpp65 for 15 min. PBMCs stimulated with CMVpp65 demonstrated an increase in the percent of CD3^+^CD4^+^pZap70^+^ cells that did not differ significantly when PBMCs were cultured with HIV DR^hi^ cells or MDSC (PBMCs 18.3 ± 1% vs DR^hi^ 13.3 ± 3.2%, p = 0.7; PBMCs 18.3 ± 1% vs MDSC 12.7 ± 3.3%, p = 0.15; Fig. 5a and b). Concomitantly, MFI of pZap70 in CD3^+^CD4^+^ cells increased when PBMCs were stimulated with the CMVpp65, and this was not affected by the presence of DR^hi^ or MDSC (PBMCs 176 ± 91 vs DR^hi^ 142 ± 47, p = 0.47; PBMCs 176 ± 91 vs MDSC 164 ± 41, p = 0.73; Fig. 5c).

The PI3K/Akt pathway is important for T cell proliferation and survival. PI3K dependent pAkt is the master regulator for T cell IL-2 and IFNγ production. Given this, we sought to investigate if B7-H4 expressed on HIV MDSC would inhibit pAkt in response to CMVpp65. Since, an optimum increase in percentage CD3^+^CD4^+^pAkt^+^ cells was observed in CMV(+) donors, 15–30 min post-stimulation with CMVpp65 (Supplemental Figure S2A Lower Panel & S2B), we studied the effect of HIV MDSC on CMVpp65 induced pAkt at 15 min post-stimulation. HIV expanded DR^hi^ and MDSC from CMV(+) HIV-uninfected donors were isolated using flow cytometry. Control or B7-H4 siRNA transfected MDSC and autologous PBMCs were co-cultured with or without CMVpp65, and pAkt was determined. No significant difference was observed in the percentage of CD3^+^CD4^+^pAkt^+^ cells stimulated with CMVpp65 compared to DR^hi^ (26 ± 3% vs 23 ± 3% p = 0.38; Fig. 5d and 5e). However, culture of PBMCs with HIV MDSC transfected with control siRNA significantly decreased the CMVpp65 induced percentage of CD3^+^CD4^+^pAkt^+^ cells (26 ± 3% vs 5.3 ± 0.3% p = 0.01). Conversely, when MDSC were transfected with B7-H4 siRNA the percentage of CD3^+^CD4^+^pAkt^+^ cells recovered when cultured with PBMCs in the presence of CMVpp65 (5.3 ± 0.3 vs 21.3 ± 2.6%; p = 0.01; Fig. 5e). Concomitantly, MFI of pAkt in CD3^+^CD4^+^ cells increased when PBMCs were stimulated with CMVpp65 and this was not affected by the presence of DR^hi^ cells (108 ± 24 vs 102 ± 31.2; p = 0.32). Culture of PBMCs with HIV MDSC transfected with control siRNA significantly decreased CMVpp65 induced MFI of pAkt in CD3^+^CD4^+^ cells (108 ± 24 vs 29.3 ± 13; p = 0.01), and this increased when MDSC were transfected with B7-H4 siRNA and cultured with PBMCs in presence of CMVpp65 (29.3 ± 13 vs 92 ± 19; p = 0.001; Fig. 5f). Collectively, these results establish that B7-H4 present on HIV MDSC plays an immune regulatory role in controlling T cell activation and subsequent IFNγ mediated inflammation in response to CMVpp65.

### IL-27 and HIV-1 infection

**Figure 6:**
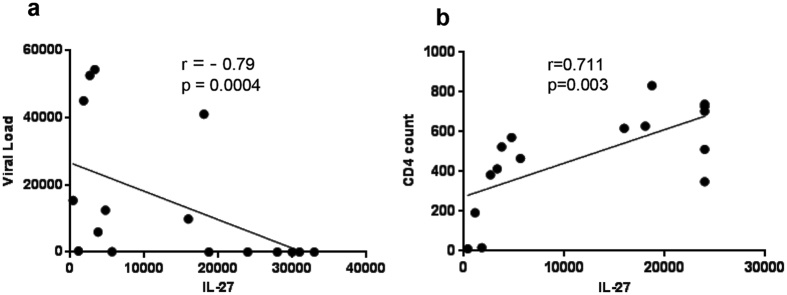
IL-27 and HIV infection. To determine the role of IL-27 during HIV infection, IL-27 was quantitated in the plasma of HIV(+) individuals (n = 16) by ELISA. **(a)** Correlation with plasma viral load (copies/mL). **(b)** Correlation of IL-27 with CD4^+^ T cell number (cells/mm^3^). For both graphs each dot represents an individual donor.

HIV infection is characterized by increased cytokine response leading to immune activation and HIV replication. In the next set of experiments, we hypothesized that successful ART not only controls viral replication, but also induces the production of immune regulatory cytokines that control inflammation and immunopathology^19^,^21^,^26^,^27^. To this end, we investigated the role of IL-27 during HIV infection and quantified the amount of viral RNA in the plasma of 16 HIV-infected individuals with and without viral suppression (Supplemental Table SI). The quantity of IL-27 in the plasma negatively correlated with viral load (r = −0.79; p = 0.0004; Fig. 6a), but showed a positive correlation with CD4^+^ T cell count (r = 0.711; p = 0.003; Supplemental Table I and Fig. 6b) further establishing the protective effect of IL-27 associated with HIV immune reconstitution. These *ex vivo* results suggest that the immune regulatory cytokine IL-27 is associated with HIV and CD4^+^ T cell recovery.

### IL-27 regulates CD4^+^ T cell IFNγ production in CMV(+) individuals

**Figure 7:**
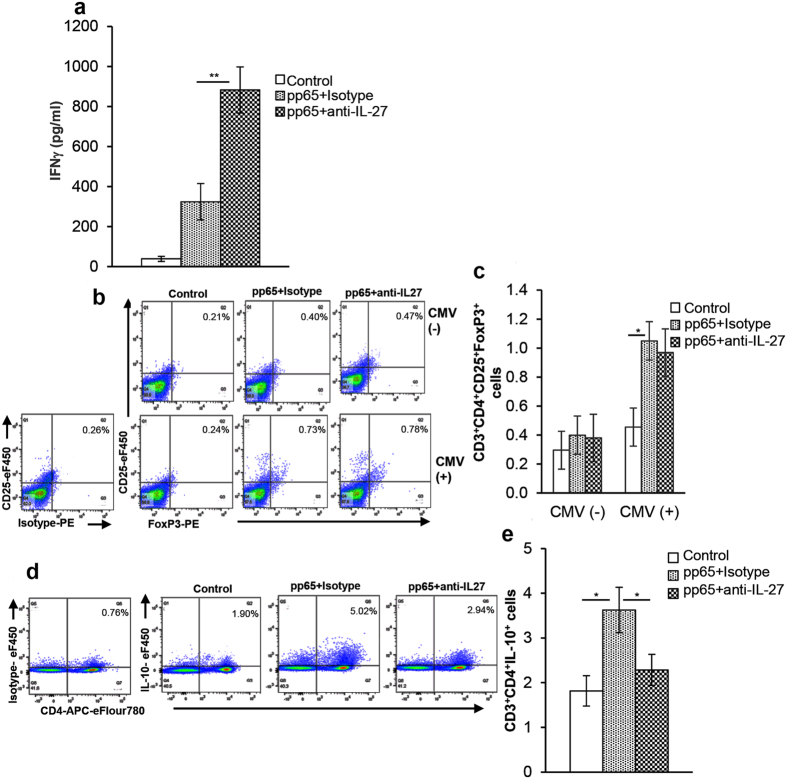
IL-27 regulates CD4^+^ T cell IFNγ production during CMV infection. **(a)** To determine the effect of IL-27 on CMV IFNγ production, PBMCs of CMV(+)/HIV(+) donors (n = 7) were cultured with or without CMVpp65 in the presence of neutralizing anti-IL-27 or matched isotype antibody. Supernatants were collected after 48 hrs and the quantity of IFNγ determined by ELISA. **(b and c)** Effect of IL-27 on Treg cell expansion was determined. PBMCs (n = 4) CMV(+) or CMV(−) HIV(+) donors were cultured with or without CMVpp65 in the presence of neutralizing anti-IL27 or matched isotype antibody. After 3 days, cells were surface stained using anti-CD3, -CD4, -CD25 Abs followed by intracellular staining using anti-FoxP3 or isotype control antibody; %CD3^+^CD4^+^CD25^+^FoxP3^+^ cells was determined by flow cytometry. (**b**) Representative flow cytometry plot from CMV(−) *(upper panel)* and CMV(+) *(lower panel)* HIV-infected individual is shown. (**c**) Histograms are presented as mean+/−SD. **(d and e)** Effect of IL-27 on IL-10 production by CD4^+^ T cells was determined. PBMCs of CMV(+) HIV(+) (n = 9) were cultured with or without the CMVpp65 in the presence of neutralizing anti-IL-27 or matched isotype antibody for 48 hrs. Brefeldin A was added for the last 5 hrs of culture. Cells were surface stained with anti-CD14, -CD3, -CD4, fixed, permeabilized and stained with anti-IL-10 for intracellular IL-10; %CD14^−^CD3^+^CD4^+^IL-10^+^ cells was determined by flow cytometry. (**d**) Representative flow cytometry plot is shown. (**e**) Histograms are presented as mean+/−SD. *p < 0.05, **p < 0.005.

Previous studies demonstrating that IL-27 limits IFNγ mediated immune pathology in various pathological conditions, prompted us to investigate if IL-27 regulates IFNγ production in response to CMV. We found that PBMCs from CMV(+) HIV-infected individuals when cultured with CMVpp65 released significantly greater quantities of IFNγ in culture supernatants compared to controls (324 ± 91 vs 39 ± 13 pg/ml, p = 0.03); this was further increased when cells were cultured in the presence of anti-IL-27 and stimulated with CMVpp65 (883 ± 115 vs 324 ± 91 pg/ml; p = 0.003; Fig. 7a). Similar findings were observed in CMV(+) HIV-uninfected donors (Supplemental Figure S3C). These findings suggest that IL-27 also regulates CMV IFNγ in the setting of HIV/CMV dual infection.

Recently, Foxp3^+^ Tregs have been shown to play an important role in the development of CMV EOD in HIV-infected individuals^28^. Therefore, we next examined if IL-27 restrict IFNγ production by mediating FoxP3^+^ cell expansion. Similar to CMV(+) HIV-uninfected donors (Supplemental Figure S3A and S3D), PBMCs from CMV(+) or CMV(−) HIV-infected subjects were stimulated with CMVpp65 in presence or absence of anti-IL27 antibody and FoxP3^+^cell expansion was determined by flow cytometry. CMVpp65 induced greater FoxP3^+^ cell expansion in CMV(+) individuals when compared to controls (1.1 ± 0.3% vs 0.5 ± 0.2%; p = 0.003) or CMV(−) donors, and this was not affected when cells were treated with anti-IL-27 (1.1 ± 0.3% vs 1 ± 0.3%; p = 0.51; Fig. 7b and c).

Having found that IL-27 does not mediate CMV FoxP3^+^ cell expansion, we next examined if IL-27 modulates IL-10 production from CD4^+^ T cells in HIV-infected and -uninfected CMV(+) subjects. Similar to CMV(+) HIV-uninfected donors (Supplemental Figure S3B and S3E), PBMCs from HIV-infected CMV(+) individuals when stimulated with CMVpp65 exhibited an increase in the percentage of CD4^+^IL-10^+^ T cells compared to controls (3.63 ± 2.1% vs 1.8 ± 1.0%; p = 0.009); cells stimulated with CMVpp65 in the presence of anti-IL-27 had a decreased percentage of CD4^+^IL-10^+^ cells (3.63 ± 2.1% vs 2.3 ± 1.0%; p = 0.014; Fig. 7d and e). Collectively, these results establish that IL-27 controls IFNγ and regulates IL-10 production from CD4^+^ T cell in individuals infected with CMV with or without HIV.

### IL-27 regulates B7-H4 expression on HIV expanded MDSC

**Figure 8:**
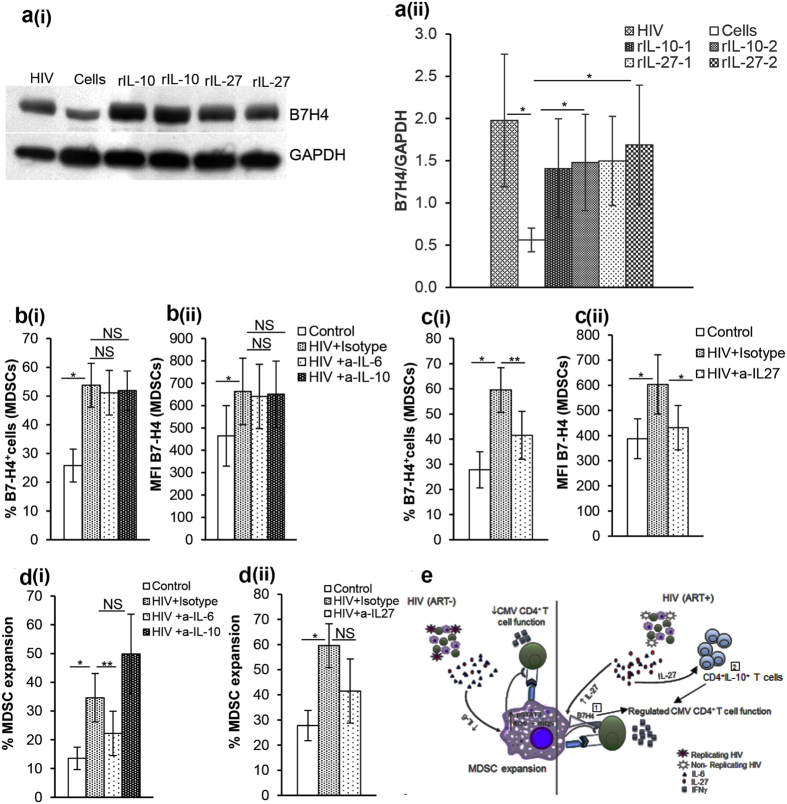
IL-27 regulates B7-H4 expression on HIV expanded MDSC. **(a)** To determine if IL-27 regulates B7-H4 expression, PBMCs from HIV(−) donors were cultured with or without rIL-27 (5 ng/ml) for 72 hrs. Cells cultured in the presence of non-infectious HIV_BaL_ (p24, 12 ng/10^7^ cells) or rIL-10 (5 ng/ml) served as additional controls. Whole cell lysates of PBMCs were prepared and immunoblotted with Abs to GAPDH as loading control and B7-H4. **(ai)** Immunoblot shown is representative of 4 donors; cells cultured with rIL10 and rIL-27 were run in duplicates. **(aii)** The histogram bar graph shows mean+/−SD of the densitometric analysis for B7-H4 (n = 4). **(b–d)** Effect of IL-6, IL-10 and IL-27 on MDSC and B7-H4^+^ HIV MDSC was determined. PBMCs of healthy donors were cultured with or without non-infectious HIV_BaL_ (p24, 12 ng/10^7^ cells) in the presence or absence of neutralizing anti-IL6, -IL-10 or –IL-27 or respective isotype antibodies for 5 days. Cells were stained with anti-CD11b, -CD33, -CD14, -HLA DR and –B7-H4 Abs and analyzed using flow cytometry. **(b)** %B7-H4^+^MDSC (bi) and MFI of B7-H4 on MDSC (bii) is shown in presence of anti-IL-6 or-IL-10 antibodies. **(c)** %B7-H4^+^MDSC (ci) and MFI of B7-H4 on MDSC (cii) is shown in presence of anti-IL-27 antibody. **(d)** %MDSC is shown in presence of anti-IL-6 and –IL-10 (di) and anti-IL-27 (dii) antibodies. Histograms are presented as mean+/−SD; n = 4 donors. **(e)** Regulation of immunity in HIV/CMV co-infection: HIV/CMV co-infected individuals on ART with suppressed HIV replication and recovered CD4^+^ T cell numbers have increased IL-27 and reduced MDSC, IL-27 induces (1) B7-H4 on MDSC which binds to CMV-CD4^+^ T cells and (2) mediates CD4^+^IL-10^+^ T cells, these collectively regulates T cell activation such that IFNγ is produced that is sufficient to control CMV replication without inducing inflammation *(right side of straight line)*. HIV/CMV co-infected ART naïve individuals with replicating virus have increased IL-6 that induces MDSC expansion and MDSC contributes to immune suppression *(left side of straight line)* observed during chronic disease stage. *p < 0.05, **p < 0.005.

Having found that B7-H4^+^ HIV MDSC and IL-27 control CMVpp65 induced IFNγ production, we hypothesized that IL-27 contributes to HIV MDSC mediated control of CMV induced IFNγ production through the induction of B7-H4 expression. To test this hypothesis, PBMCs were cultured with rIL-27 for 72 hrs and B7-H4 expression in total cell protein was analyzed by immunoblotting. Since IL-10 is known to induce B7-H4 expression on various APC^29^,^30^, we used PBMCs cultured with rIL-10 as a control. To detect B7-H4 expression during HIV infection, PBMCs cultured with non-infectious HIV were used as an additional control. B7-H4 expression was increased in cells cultured with non-infectious HIV or rIL-10 compared to control cells (Fig. 8ai and aii). Importantly, rIL-27 also increased the expression of B7-H4 in PBMCs compared to controls (Fig. 8ai and aii). We next investigated if IL-10 and IL-27 regulate B7-H4 on HIV MDSC. Previously, we have shown that MDSC expansion in HIV infection is regulated by the autocrine production of IL-6^12^. Therefore, we investigated if IL-6 also modulates B7-H4 expression on HIV MDSC. PBMCs from healthy donors were cultured with or without non-infectious HIV in the presence or absence of neutralizing anti-IL-27, -IL-6 or -IL-10 Ab; B7-H4 on MDSC and DR^hi^ cells were identified. Consistent with our earlier observation, the percent of B7-H4^+^MDSC^+^ cells was increased in PBMCs cultured with HIV compared to control cells (53.8 ± 7.6 vs 25.8 ± 6;p = 0.02) or DR^hi^ cells (data not shown). Neutralizing IL-6 or IL-10 had no effect on B7-H4^+^ MDSC (53.8 ± 7.6 vs 52 ± 6.8 and 51.1 ± 7.8, p = 0.31 and 0.18, respectively) (Fig. 8bi). Importantly, the presence of anti-IL-27 decreased the percentage of B7-H4^+^MDSC^+^ cells (60 ± 8.9 vs 41.5 ± 9.5; p = 0.002) (Fig. 8ci). Similarly, B7-H4 MFI on HIV MDSC was high compared to control cells (663.3 ± 149 vs 464 ± 135; p = 0.04) and DR^hi^ cells (data not shown); anti-IL-6 or –IL-10 had no effect on B7-H4 MFI (663.3 ± 149 vs 650.7.5 ± 148.6 and 641 ± 143.6, p = 0.3 and 0.3, respectively) (Fig. 8bii). The presence of anti-IL-27 decreased B7-H4 MFI on HIV MDSCs (603.5 ± 118 vs 431.7 ± 88.1; p = 0.03) (Fig. 8cii). As expected, culture of PBMCs with HIV resulted in MDSC expansion (13.5 ± 3.9 vs 34.6 ± 8.4; p = 0.02), and this was inhibited by the presence of anti-IL-6 Ab (34.6 ± 8.4 vs 22.2 ± 7.7; p = 0.003) (Fig. 8di). The presence of anti- IL-10 antibody had no effect on MDSC expansion (34.6 ± 8.4 vs 49.8 ± 13.9, p = 0.31) (Fig. 8di). The presence of anti-IL-27 also did not affect HIV MDSC expansion (50.7 ± 8.7 vs 53 ± 12.7; p = 0.42) (Fig. 8dii). Collectively, these results suggest that although IL-6 mediates MDSC expansion, it is not involved in B7-H4 mediated immune regulation. In contrast, although IL-27 fails to promote MDSC expansion, it induces B7-H4 expression on MDSC which regulates immune activation during HIV infection.

## Discussion

Although CMV EOD has been greatly reduced in the ART era, CMV is still implicated in persistent immune activation and increased risk of CVD in persons with sustained HIV suppression^10^,^31^. The data presented here demonstrate that although CD4^+^ T cells from HIV-infected persons on ART produce less IFNγ in response to CMVpp65, there is a significantly increased frequency of CD4^+^CX3CR1^+^IFNγ^+^ cells which are associated with increased endothelial cell inflammation and CVD^7^,^8^,^10^. Additionally, we demonstrate to our knowledge for the first time, that HIV MDSC can control CMVpp65 induced immune activation through mechanisms involving B7-H4 and IL-27. Previously, we and others have shown increased numbers and frequency of MDSC in untreated HIV-infected individuals^12^,^32^. In the present study, we found MDSC frequency in HIV-infected individuals with suppressed viral replication remains high compared to uninfected controls. This supports previous findings^32^,^33^ which have shown that MDSC numbers decline with ART but remain high compared to HIV-uninfected controls.

MDSC expand during various pathological conditions due to acute or chronic inflammation and are an important immunosuppressive factor^12^,^15^,^34^. Although regarded as detrimental for immunity, adoptive transfer of MDSC ameliorates the severity of inflammatory diseases by regulating CD4^+^ T cells, T_H_17 cells, inflammatory cytokines and expanding IL-10 and TGFβ^35^,^36^. These divergent roles suggest that the differential effects of MDSC depend on the disease condition, timing and severity of the inflammatory response. HIV infection is characterized by cytokine burst leading to excessive inflammation and promoting MDSC expansion. The increase in MDSC accompanied by CD4^+^ T cell depletion further magnifies the immune suppression. However, as the HIV viral load declines and CD4^+^ T cells recover, MDSC serve as immune regulators, controlling exuberant production of IFNγ by CMV-specific CD4^+^ T cells.

Despite the importance of CMV-CD4^+^ T cells in controlling CMV viremia, kinetic studies performed in the setting of HIV/CMV co-infection have shown that CMV-CD4^+^ T cells and CMV-IFNγ increase after 3–4 weeks of ART initiation and start to decline thereafter^9^,^37^,^38^. Although the threshold of T cell activation to control CMV replication is not well-defined, a low number of CMV-CD4^+^ T cells are sufficient to control CMV reactivation. However, IFNγ produced by the CX3CR1^+^CD4^+^ activated T cells can lead to endothelial cell inflammation and increased risk for CVD^7^,^8^,^11^. In the setting of HIV/CMV co-infection, CX3CR1^+^ CMV CD4^+^ T cells are CD27^−^, CD57^+^, PD1^+^ and major producers of IFNγ^10^ (and Garg *et al*. unpublished observations) indicative of pro-inflammatory T cells. Similar to a recent study of Pachino *et al*., we also observed that CMV specific CD4^+^CX3CR1^+^ cells expressed cytotoxic molecules perforin, granzyme B^39^ and transcription factor EOMES (data not shown). These findings collectively suggest that CMV infection can potentiate vascular tissue damage and CD4^+^CX3CR1^+^ cells may be a target for therapeutic intervention. In the present study, we provide evidence that HIV MDSC are able to control CMV mediated immune activation by restricting CMV specific CX3CR1^+^CD4^+^ IFNγ^+^ T cells and contributing to IL-10 production.

PD-1/PD-L1 or PD-L2 interaction causes reversible T cell dysfunction and exhaustion in HIV-infected individuals^40^,^41^. Similar to the previous findings, we also observed increased expression of PD-L1 and PD-L2 on HIV expanded DR^hi^ and MDSC^41^; however, this was not characteristic of HIV MDSC. IL-6 mediates HIV MDSC expansion, but does not modulate B7-H4 expression. Nonetheless, IL-27 did not mediate MDSC expansion, but regulated the expression of B7-H4 on HIV MDSC. This is of significance during HIV infection where HIV replication and elevated IL-6 are associated with increased MDSC numbers. In HIV-infected persons on ART, IL-6 decreases with a corresponding decline in MDSC and an increase in IL-27. Thus, IL-27 plays a dominant role in regulating immune activation through B7-H4 (Fig. 8e). We (data not shown) and others^42^,^43^ observed that rIL-27 directly inhibits HIV replication in PBMC. Although the mechanism of viral suppression is not fully established, it appears that the inhibition of spectrin β nonerythrocyte 1 (SPTBN1) by IL-27 plays an important role^42^.

Uncontrolled T cell responses exacerbate inflammatory disorders which may be controlled by the administration of B7-H4 mAb^25^,^44^,^45^. In this research, we show that B7-H4 expressed on HIV MDSC controls CMV induced T cell activation in HIV/CMV co-infection conditions. B7-H4 ligation to CMV antigen primed T cells does not affect early events of TCR signaling (pZap70), but rather inhibits pAkt which is known to be regulated by the CD28 co-receptor as also seen by Wang *et al*.^25^. In contrast to blocking B7-H4 in order to potentiate T cell activity and cancer prognosis, we propose that in HIV/CMV co-infected individuals following immune reconstitution, B7-H4 serves as regulator of immune activation without compromising CMV immunity. However, in HIV/CMV co-infected individuals with low CD4^+^ T cells, B7-H4/MDSC is involved in progressive immune suppression (Fig. 8e).

IL-27 utilizes multiple mechanisms to control inflammation associated with T_H_1, T_H_2 and T_H_17 cells. Similar to studies using IL-27R^−/−^ mice^21^,^27^ we also observed that IL-27 neutralization increased the production of CMV IFNγ in HIV/CMV co-infected individuals. Unlike the findings of Hall *et al*.^20^, IL-27 neutralization did not affect the expansion of CMV induced FoxP3^+^ cells in HIV/CMV co-infected individuals. However, our finding showing that IL-27 inhibits IL-10^+^CD4^+^ T cells is consistent with those of Stumhofer *et al*.^46^. We are currently investigating the role of IL-27 in MDSC mediated control of immune activation by IL-10. Our findings that IL-27 does not affect FoxP3^+^ cell expansion but inhibits IL-10^+^CD4^+^ T cells suggests that IL-27 regulates the activity of FoxP3^−^ Treg cells, classified as Type 1 regulatory T cells (Tr1) in individuals infected with CMV with or without HIV. Importantly, in this study, we provide the first evidence of IL-27 mediated regulation of IFNγ and IL-10 during human CMV infection in the setting of HIV co-infection.

In summary, our *ex vivo* and *in vitro* data collectively establish that HIV/CMV co-infected individuals with suppressed HIV replication and recovered CD4^+^ T cells have an increased quantity of MDSC and cytokine IL-27. IL-27 induces the expression of B7-H4 on MDSC which regulates T cell pAkt and restricts subsequent T cell activation in response to CMV. In addition, we show that IL-27 controls IFNγ and mediates IL-10 producing Tr1 cells during CMV infection, with or without co-infection with HIV (Fig. 8e). These findings provide a mechanistic model of how MDSC can control CMV induced chronic inflammation and T cell activation which is associated with increased risk for CVD observed in persons on ART with sustained HIV suppression. Moreover, our findings suggest that IL-27 and B7-H4^+^ MDSC provide attractive new therapeutic targets to control persistent immune activation during HIV-infection.

## Materials and Methods

### Patient population

Blood was drawn after obtaining written informed consent from HIV seronegative and HIV-infected donors. All studies were approved by the Institutional Review Board of the Human Research Protection Program of University of California, San Diego in accordance with the requirements of the Code of Federal Regulations on the Protection of Human Subjects (21 CFR Parts 50 and 56, and 45 CFR 46).

### Cell isolation and culture

Peripheral blood mononuclear cells (PBMCs) were isolated from freshly obtained blood by Ficoll density centrifugation (GE Healthcare, Uppsala) and cultured in RPMI 1640 (Gibco) and 10% human serum (MP Biomedicals) in the presence or absence of heat inactivated HIV-1_BAL_ at 12 ng/10^7^ cells of p24 antigen equivalent.

### Antibodies and other reagents

Antibodies used for flow cytometry were Alexa Fluor488-anti-CD11b, PerCP-eF710/PE/or PE-Dazzel594-anti-HLA DR (clone L243), eF450-anti-HLA DR (clone LN3) PE/Cy7-anti-CD14, APC-anti-CD33, BV510-anti-CD3, BV605-anti-CD19, PerCP-Cy5.5-anti-CD66b, Alexa Fluor700 or PerCP-Cy5.5-anti-IFNγ, APC/Cy7-CD38, PerCP-Cy5.5-anti-CD69, PE-anti-CX3CR1, -anti-B7-H4, -anti-PD-L1, -anti-B7-H3, -anti-PD-L2, -anti-ICOS-L (all from Biolegend, San Diego, CA); FITC- or APCeF780-anti-CD4, Alexa Fluor700- or eF450-anti-CD25, PE-anti-FoxP3 and eF450-anti-IL10 (all from eBioscience); PE-anti- phospho-Zap70^(pY292)^, PE-anti-phospho-Akt^(pS473)^ from BD Biosciences. For neutralization studies, mAb to IL-10, IL-6 or rat IgG isotype (10 μg/mL, Biolegend) and polyclonal goat anti-IL-27 or normal goat IgG control (10 μg/mL R&D Systems) control were used.

### Isolation of CD11b^+^CD33^+^CD14^+^HLA DR^hi^ (DR^hi^) and CD11b^+^CD33^+^CD14^+^HLA DR^−/lo^ (MDSC) cells

PBMCs from CMV seropositive HIV seronegative healthy donors (HIV-negative) were cultured with heat inactivated HIV for 5 days, stained for CD11b, CD33, CD14, HLA DR. DR^hi^ and MDSC and isolated using MoFlo flow cytometer; sorted cells were >98% positive.

### Depletion of MDSC from fresh PBMCs

Freshly isolated PBMCs were stained for CD14 and HLA DR (clone L243); CD14^+^HLA DR^−/lo^ MDSC were depleted using flow cytometry. MDSC and MDSC depleted PBMC fractions were further stained for CD11b, CD33, CD3, CD19 and CD66b (MDSC) and CD3 and HLA DR (clone LN3) (MDSC depleted PBMCs).

### Immunolabelling and flow cytometry

Cells were surface stained for surface markers using respective antibodies and cell staining buffer (Biolegend). For intracellular phosphorylated (p)- Zap70 (pZap70) and Akt (pAkt), cells were fixed with Fixation Buffer and permeabilized with Phosphoflow Perm Buffer III (both from BD Biosciences) followed by staining with PE–anti-pZap70^(pY292)^ or –anti-pAkt^(pS473)^. PBMCs were stained for intracellular FoxP3, IFNγ and IL10 using Cytofix/Cytoperm Plus kit and anti-FoxP3, -IFNγ and -IL10 (all from eBioscience). Flow cytometry was done on FACS Canto and data analyzed using Flowjo (Treestar). Controls for each experiment included unstained cells, fluorescence minus one (FMO) and isotype matched antibodies.

### B7-H4 silencing

B7-H4 in sorted HIV MDSC was knocked down by transfecting scrambled or B7-H4 small interfering RNA (siRNA) (Smartpool, Thermo Scientific) using Lipofectamine^TM^ RNAiMAX (Invitrogen) as per manufacturer’s instructions. Nucleotide BLAST was performed for each siRNA present in the siRNA pool; it did not show homology to any other B7-member of proteins. Silencing was evaluated by preparing cell lysates and immunoblotting for B7-H4 and GAPDH. Cells transfected with scrambled siRNA were used as negative control.

### Co-culture of MDSC or DR^hi^ cells with PBMCs

For Enzyme-Linked ImmunoSpot Assay (ELISPOT), 1 × 10^5^ sorted MDSC or HLD DR^hi^ cells were co-cultured with autologous 2 × 10^5^ PBMCs in the presence of 1 μg/ml peptide pool of CMVpp65 (CMVpp65) (NIH AIDS Reagent Program) for 18–20 hrs. For ELISA and intracellular staining, 2 × 10^5^ sorted MDSC or HLD DR^hi^ cells were co-cultured with autologous 4 × 10^5^ PBMCs in the presence of CMVpp65 for 48 hrs, supernatants were frozen and cells stained for intracellular IFNγ. For ELISA and pAkt staining using B7-H4 silenced MDSC, 25,000 MDSC were cultured with 50,000 PBMCs and stimulated with CMVpp65. Cells were stained following 30 min of stimulation to assess pAkt. Supernatants were frozen for IL-2 and IFNγ quantitation. For some experiments, 0.05 × 10^6^ sorted MDSC were co-cultured with 0.1 × 10^6^ autologous PBMCs in presence of CMVpp65 with or without recombinant B7-H4 (rB7-H4; 10 ng/ml, R&D Systems) for 18–48 hrs. Supernatants were frozen for IL-2 and IFNγ quantitation.

### ELISPOT

Frequencies of IFNγ or IL-10 producing cells were determined in PBMC-sorted DR^hi^ or MDSC co-cultured in ELISPOT plates coated with capture antibodies for IFNγ and IL-10, respectively. Cytokine producing cells were detected using kits for IFNγ (BD Biosciences) and IL-10 (Mabtech) according to manufacturer’s instructions. Air-dried spots were counted with automated reader S5 UV Lite Analyzer (Immunospot). For some experiments, PBMC from CMV(+) HIV-infected or HIV-uninfected donors were cultured in the presence of 1 μg/ml of CMVpp65 for 48–72 hrs, CD4^+^CX3CR1^−^ and CD4^+^CX3CR1^+^ cells were sorted and cultured in ELISPOT plates coated with capture antibody for IFNγ. Cytokine producing cells were detected as above.

### Quantification of cytokines

Supernatants from PBMCs and sorted cell co-cultures were used to determine levels of IFNγ ELISA (Biolegend), IL-2HS (sensitivity 0.4 pg/ml) and IFNγHS (sensitivity 0.99 pg/ml) (both from eBioscience). Plasma IL-27 levels were measured using Duoset ELISA kit (R&D Systems).

### Immunoblotting

Immunoblotting of cell lysates was performed as previously described^47^ and relative densities for target protein bands to housekeeping GAPDH bands were compared using ImageJ (NIH).

### Statistical analysis

Data are expressed as mean values ± standard deviation. Paired Student’s *t*-tests were used to determine the statistical significance for *in vitro* experiments. Comparisons between HIV-infected and –uninfected subjects was made by non-parametric Mann Whitney U test; comparisons between different parameters were analyzed using Spearman correlation. For data that were not normally distributed, the Wilcoxon rank-sum test was used. Statistical analysis was performed using Graphpad Prism 5 (La Jolla, CA). A p-values < 0.05 were considered statistically significant.

## Additional Information

**How to cite this article:** Garg, A. *et al*. Human Immunodeficiency Virus Type-1 Myeloid Derived Suppressor Cells Inhibit Cytomegalovirus Inflammation through Interleukin-27 and B7-H4. *Sci. Rep.* **7**, 44485; doi: 10.1038/srep44485 (2017).

**Publisher's note:** Springer Nature remains neutral with regard to jurisdictional claims in published maps and institutional affiliations.

## Supplementary Material

Supplemental Information

## References

[b1] Anglaret X. . AIDS and non-AIDS morbidity and mortality across the spectrum of CD4 cell counts in HIV-infected adults before starting antiretroviral therapy in Cote d’Ivoire. Clin Infect Dis 54, 714–723, doi: 10.1093/cid/cir898 (2012). 22173233 PMC3275759

[b2] Saharia K. K. & Koup R. A. T cell susceptibility to HIV influences outcome of opportunistic infections. Cell 155, 505–514, doi: 10.1016/j.cell.2013.09.045 (2013). 24243010 PMC3858849

[b3] Bronke C. . Dynamics of cytomegalovirus (CMV)-specific T cells in HIV-1-infected individuals progressing to AIDS with CMV end-organ disease. The Journal of infectious diseases 191, 873–880, doi: 10.1086/427828 (2005). 15717261

[b4] Casazza J. P. . Acquisition of direct antiviral effector functions by CMV-specific CD4+ T lymphocytes with cellular maturation. The Journal of experimental medicine 203, 2865–2877, doi: 10.1084/jem.20052246 (2006). 17158960 PMC2118179

[b5] Sylwester A. W. . Broadly targeted human cytomegalovirus-specific CD4+ and CD8+ T cells dominate the memory compartments of exposed subjects. The Journal of experimental medicine 202, 673–685, doi: 10.1084/jem.20050882 (2005). 16147978 PMC2212883

[b6] Gamadia L. E. . Primary immune responses to human CMV: a critical role for IFN-gamma-producing CD4+ T cells in protection against CMV disease. Blood 101, 2686–2692, doi: 10.1182/blood-2002-08-2502 (2003). 12411292

[b7] Bolovan-Fritts C. A., Trout R. N. & Spector S. A. Human cytomegalovirus-specific CD4+ -T-cell cytokine response induces fractalkine in endothelial cells. Journal of virology 78, 13173–13181, doi: 10.1128/JVI.78.23.13173-13181.2004 (2004). 15542669 PMC525022

[b8] Bolovan-Fritts C. A., Trout R. N. & Spector S. A. High T-cell response to human cytomegalovirus induces chemokine-mediated endothelial cell damage. Blood 110, 1857–1863, doi: 10.1182/blood-2007-03-078881 (2007). 17519388 PMC1976357

[b9] Hsu D. C. . Restoration of CMV-specific-CD4 T cells with ART occurs early and is greater in those with more advanced immunodeficiency. PloS one 8, e77479, doi: 10.1371/journal.pone.0077479 (2013). 24130889 PMC3795037

[b10] Sacre K. . A role for cytomegalovirus-specific CD4+ CX3CR1+ T cells and cytomegalovirus-induced T-cell immunopathology in HIV-associated atherosclerosis. Aids 26, 805–814, doi: 10.1097/QAD.0b013e328351f780 (2012). 22313962 PMC4155398

[b11] van de Berg P. J., Yong S. L., Remmerswaal E. B., van Lier R. A. & ten Berge I. J. Cytomegalovirus-induced effector T cells cause endothelial cell damage. Clinical and vaccine immunology: CVI 19, 772–779, doi: 10.1128/CVI.00011-12 (2012). 22398244 PMC3346330

[b12] Garg A. & Spector S. A. HIV type 1 gp120-induced expansion of myeloid derived suppressor cells is dependent on interleukin 6 and suppresses immunity. The Journal of infectious diseases 209, 441–451, doi: 10.1093/infdis/jit469 (2014). 23999600 PMC3883171

[b13] Condamine T. & Gabrilovich D. I. Molecular mechanisms regulating myeloid-derived suppressor cell differentiation and function. Trends in immunology 32, 19–25, doi: 10.1016/j.it.2010.10.002 (2011). 21067974 PMC3053028

[b14] Fujimura T., Ring S., Umansky V., Mahnke K. & Enk A. H. Regulatory T cells stimulate B7-H1 expression in myeloid-derived suppressor cells in ret melanomas. The Journal of investigative dermatology 132, 1239–1246, doi: 10.1038/jid.2011.416 (2012). 22189788

[b15] Gabrilovich D. I. & Nagaraj S. Myeloid-derived suppressor cells as regulators of the immune system. Nature reviews. Immunology 9, 162–174, doi: 10.1038/nri2506 (2009). PMC2828349 19197294

[b16] Liu Y. . Regulation of arginase I activity and expression by both PD-1 and CTLA-4 on the myeloid-derived suppressor cells. Cancer immunology, immunotherapy: CII 58, 687–697, doi: 10.1007/s00262-008-0591-5 (2009). 18828017 PMC11030939

[b17] Hall A. O., Silver J. S. & Hunter C. A. The immunobiology of IL-27. Advances in immunology 115, 1–44, doi: 10.1016/B978-0-12-394299-9.00001-1 (2012). 22608254

[b18] Yoshida H. & Miyazaki Y. Regulation of immune responses by interleukin-27. Immunological reviews 226, 234–247, doi: 10.1111/j.1600-065X.2008.00710.x (2008). 19161428

[b19] Fitzgerald D. C. . Suppression of autoimmune inflammation of the central nervous system by interleukin 10 secreted by interleukin 27-stimulated T cells. Nature immunology 8, 1372–1379, doi: 10.1038/ni1540 (2007). 17994023

[b20] Hall A. O. . The cytokines interleukin 27 and interferon-gamma promote distinct Treg cell populations required to limit infection-induced pathology. Immunity 37, 511–523, doi: 10.1016/j.immuni.2012.06.014 (2012). 22981537 PMC3477519

[b21] Liu F. D. . Timed action of IL-27 protects from immunopathology while preserving defense in influenza. PLoS pathogens 10, e1004110, doi: 10.1371/journal.ppat.1004110 (2014). 24809349 PMC4014457

[b22] Guzzo C., Hopman W. M., Che Mat N. F., Wobeser W. & Gee K. Impact of HIV infection, highly active antiretroviral therapy, and hepatitis C coinfection on serum interleukin-27. Aids 24, 1371–1374, doi: 10.1097/QAD.0b013e3283391d2b (2010). 20375875

[b23] Guzzo C., Hopman W. M., Che Mat N. F., Wobeser W. & Gee K. IL-27-induced gene expression is downregulated in HIV-infected subjects. PloS one 7, e45706, doi: 10.1371/journal.pone.0045706 (2012). 23049843 PMC3458084

[b24] Libby P. Inflammation in atherosclerosis. Nature 420, 868–874, doi: 10.1038/nature01323 (2002). 12490960

[b25] Wang X. . B7-H4 Treatment of T Cells Inhibits ERK, JNK, p38, and AKT Activation. PloS one 7, e28232, doi: 10.1371/journal.pone.0028232 (2012). 22238573 PMC3251556

[b26] Villarino A. . The IL-27R (WSX-1) is required to suppress T cell hyperactivity during infection. Immunity 19, 645–655 (2003). 14614852 10.1016/s1074-7613(03)00300-5

[b27] Villegas-Mendez A. . IL-27 receptor signalling restricts the formation of pathogenic, terminally differentiated Th1 cells during malaria infection by repressing IL-12 dependent signals. PLoS pathogens 9, e1003293, doi: 10.1371/journal.ppat.1003293 (2013). 23593003 PMC3623720

[b28] Weinberg A. . Regulatory T cells and the risk of CMV end-organ disease in patients with AIDS. J Acquir Immune Defic Syndr 66, 25–32, doi: 10.1097/QAI.0000000000000095 (2014). 24378728 PMC3981937

[b29] Kryczek I. . Cutting edge: induction of B7-H4 on APCs through IL-10: novel suppressive mode for regulatory T cells. Journal of immunology 177, 40–44 (2006). 10.4049/jimmunol.177.1.40 16785496

[b30] Kryczek I. . B7-H4 expression identifies a novel suppressive macrophage population in human ovarian carcinoma. The Journal of experimental medicine 203, 871–881, doi: 10.1084/jem.20050930 (2006). 16606666 PMC2118300

[b31] Hsue P. Y. . Increased carotid intima-media thickness in HIV patients is associated with increased cytomegalovirus-specific T-cell responses. Aids 20, 2275–2283, doi: 10.1097/QAD.0b013e3280108704 (2006). 17117013

[b32] Qin A. . Expansion of monocytic myeloid-derived suppressor cells dampens T cell function in HIV-1-seropositive individuals. Journal of virology 87, 1477–1490, doi: 10.1128/JVI.01759-12 (2013). 23152536 PMC3554138

[b33] Gama L. . Expansion of a subset of CD14highCD16negCCR2low/neg monocytes functionally similar to myeloid-derived suppressor cells during SIV and HIV infection. Journal of leukocyte biology 91, 803–816, doi: 10.1189/jlb.1111579 (2012). 22368280 PMC3336772

[b34] Delano M. J. . MyD88-dependent expansion of an immature GR-1(+)CD11b(+) population induces T cell suppression and Th2 polarization in sepsis. The Journal of experimental medicine 204, 1463–1474, doi: 10.1084/jem.20062602 (2007). 17548519 PMC2118626

[b35] Fujii W. . Myeloid-derived suppressor cells play crucial roles in the regulation of mouse collagen-induced arthritis. Journal of immunology 191, 1073–1081, doi: 10.4049/jimmunol.1203535 (2013). 23804709

[b36] Brudecki L., Ferguson D. A., McCall C. E. & El Gazzar M. Myeloid-derived suppressor cells evolve during sepsis and can enhance or attenuate the systemic inflammatory response. Infection and immunity 80, 2026–2034, doi: 10.1128/IAI.00239-12 (2012). 22451518 PMC3370575

[b37] Naeger D. M. . Cytomegalovirus-specific T cells persist at very high levels during long-term antiretroviral treatment of HIV disease. PloS one 5, e8886, doi: 10.1371/journal.pone.0008886 (2010). 20126452 PMC2813282

[b38] Weinberg A. . Cytomegalovirus-specific immunity and protection against viremia and disease in HIV-infected patients in the era of highly active antiretroviral therapy. The Journal of infectious diseases 193, 488–493, doi: 10.1086/499826 (2006). 16425127

[b39] Pachnio A. . Cytomegalovirus Infection Leads to Development of High Frequencies of Cytotoxic Virus-Specific CD4+ T Cells Targeted to Vascular Endothelium. PLoS pathogens 12, e1005832, doi: 10.1371/journal.ppat.1005832 (2016). 27606804 PMC5015996

[b40] Trautmann L. . Upregulation of PD-1 expression on HIV-specific CD8+ T cells leads to reversible immune dysfunction. Nature medicine 12, 1198–1202, doi: 10.1038/nm1482 (2006). 16917489

[b41] Wang X. . B7-H1 up-regulation impairs myeloid DC and correlates with disease progression in chronic HIV-1 infection. European journal of immunology 38, 3226–3236, doi: 10.1002/eji.200838285 (2008). 18924219

[b42] Dai L. . IL-27 inhibits HIV-1 infection in human macrophages by down-regulating host factor SPTBN1 during monocyte to macrophage differentiation. The Journal of experimental medicine 210, 517–534, doi: 10.1084/jem.20120572 (2013). 23460728 PMC3600911

[b43] Fakruddin J. M. . Noninfectious papilloma virus-like particles inhibit HIV-1 replication: implications for immune control of HIV-1 infection by IL-27. Blood 109, 1841–1849, doi: 10.1182/blood-2006-02-001578 (2007). 17068156 PMC1801045

[b44] Prasad D. V., Richards S., Mai X. M. & Dong C. B7S1, a novel B7 family member that negatively regulates T cell activation. Immunity 18, 863–873 (2003). 12818166 10.1016/s1074-7613(03)00147-x

[b45] Wei J., Loke P., Zang X. & Allison J. P. Tissue-specific expression of B7x protects from CD4 T cell-mediated autoimmunity. The Journal of experimental medicine 208, 1683–1694, doi: 10.1084/jem.20100639 (2011). 21727190 PMC3149222

[b46] Stumhofer J. S. . Interleukins 27 and 6 induce STAT3-mediated T cell production of interleukin 10. Nature immunology 8, 1363–1371, doi: 10.1038/ni1537 (2007). 17994025

[b47] Garg A., Rawat P. & Spector S. A. Interleukin 23 produced by myeloid dendritic cells contributes to T-cell dysfunction in HIV type 1 infection by inducing SOCS1 expression. J Infect Dis 211, 755–768, doi: 10.1093/infdis/jiu523 (2015). 25234720 PMC4402373

