# Supplementary material for: Human Immunodeficiency Virus Type-1 Myeloid Derived Suppressor Cells Inhibit Cytomegalovirus Inflammation through Interleukin-27 and B7-H4
Source: Sci Rep. 2017 Mar 24;7:44485. doi: 10.1038/srep44485 (PMC5364511; doi:10.1038/srep44485)
Supplement: Supplemental Information [file srep44485-s1.pdf]

Human Immunodeficiency Virus Type-1 Myeloid Derived Suppressor Cells Inhibit Cytomegalovirus Inflammation through Interleukin-27 and B7-H4

Ankita Garg [§], Rodney Trout[§] and Stephen A. Spector[§,†,*]

[§]Department of Pediatrics, Division of Infectious Diseases, University of California San Diego, La Jolla, California 92093-0672, USA

[†]Rady Children's Hospital, San Diego, California, 92123, USA

*Address correspondence to: *Stephen A. Spector, MD,

University of California San Diego, 9500 Gilman Drive

La Jolla, CA 92093-0672 USA

Phone: 858-534-7055 **Fax**: 858-534-7411

E-mail: saspector@ucsd.edu

Supplemental Figure S1

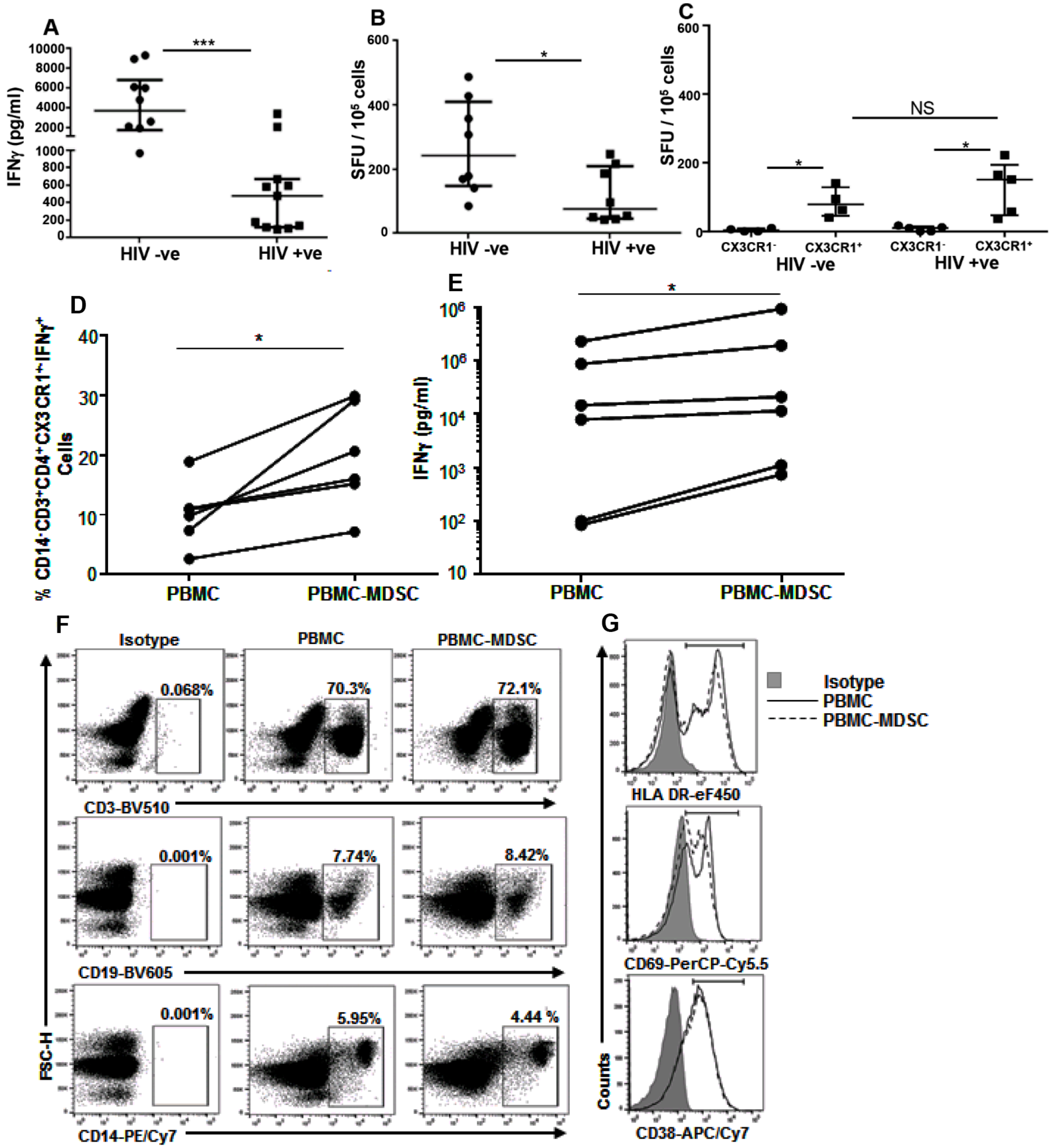

CMVpp65 specific IFNγ response is suppressed in HIV(+) donors. (A, B & C) All donors were CMV(+) and either HIV-infected (HIV+) or HIV–uninfected (HIV-). **(A)** PBMCs from HIV(-) (n=9) and HIV(+) (n=11) donors were cultured with or without peptide pool of CMVpp65 (1 μg/ml) for 72 hrs. IFNγ (pg/ml) in culture supernatants

was determined by ELISA. Net IFNγ produced was calculated: IFNγ by Control cells – IFNγ by pp65 stimulated cells. **(B)** PBMCs from 8 HIV(-) and HIV(+) donors were cultured on ELISPOT plates coated with capture anti-IFNγ Ab with or without CMVpp65. After 18 hrs, plates were washed and biotinylated anti-IFNγ detection Ab was added for 2 hrs followed by addition of Streptavidin-HRP enzyme conjugate for 1 hr; plates were washed and substrate was added using AEC substrate kit (BD Biosciences). The substrate reaction was stopped by washing wells with water. Spots were air-dried, counted on an automated ELISPOT reader and spot forming units (SFU)/10^5 PBMCs were calculated. **(C)** PBMCs from HIV(-) (n=4) and HIV(+) (n=5) donors were cultured with or without peptide pool of CMVpp65 (1 μg/ml) for 48-72 hrs. $CX3CR1^-$ and $CX3CR1^+$ cells were sorted and cultured on ELISPOT plates coated with capture anti-IFNγ Ab. After 18 hrs, cytokine producing cells were detected as above. **(D & E)** Freshly isolated PBMCs from CMV(+) HIV-uninfected healthy donors were stained with anti-CD14 and –HLA DR (clone L243) antibodies; $CD14^+$HLA $DR^{-/lo}$ MDSC from few PBMCs were depleted using flow cytometry. Whole PBMC (PBMC) and MDSC depleted PBMC (PBMC-MDSC) were cultured with or without CMVpp65 for 72 hrs with Brefeldin A added for the last 5 hrs of culture. Culture supernatant was stored and cells were surface stained with anti-CD14, -CD3, -CD4 and –CX3CR1, fixed, permeabilized and stained using anti-IFNγ for intracellular IFNγ. **(D)** Percentage of $CD14^-$ $CD3^+CD4^+CX3CR1^+IFN\gamma^+$ cells was determined by flow cytometry. **(E)** Amount of IFNγ in the culture supernatants was determined by ELISA. **(F & G)** $CD14^+$HLA $DR^{-/lo}$ MDSC were depleted from PBMCs of CMV(+) HIV-infected individuals on ART and with suppressed viral load, as above. PBMC and PBMC-MDSC were stained with **(F)** anti-CD3,- CD19 and –CD14 or isotype control antibodies and percentage of $CD3^+$, $CD19^+$ and $CD14^+$ cells was determined by flow cytometry. **(G)** anti- CD3, - HLA DR (clone LN3), -CD69 and –CD38; cells were gated on $CD3^+$ and expression of HLA DR, CD69 and CD38 was determined. Representative flow cytometry plots are shown **(A-E).** Each dot in the plots depicts data of each individual donor; **(A, B& C)**, the plots include observations from 25th to 75th percentile; the horizontal line represents the median value. *p<0.05, ***p<0.0005, NS Non significant

Supplemental Figure S2

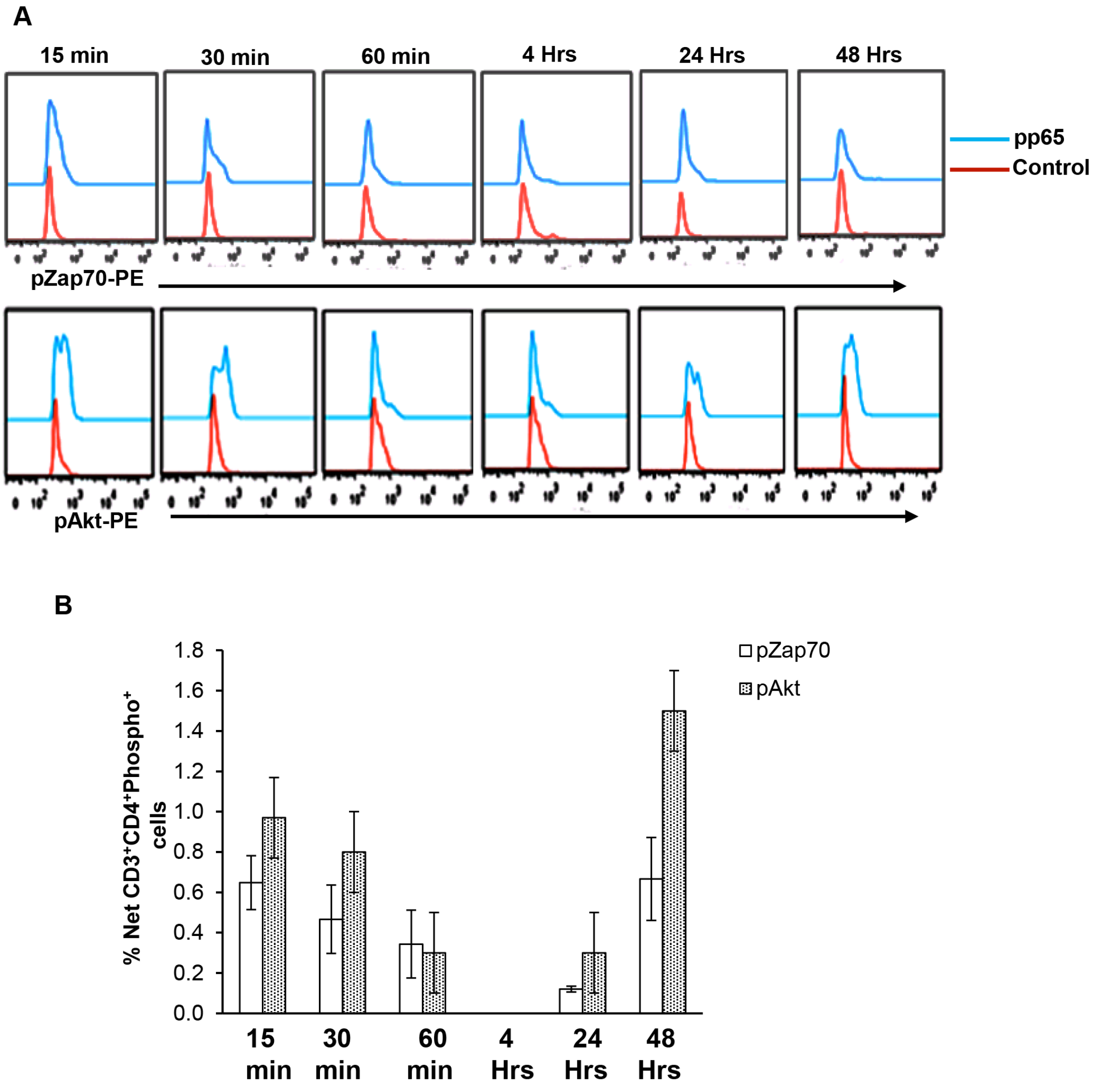

Kinetics of pZap70 and pAkt. To determine the kinetics of activation of Zap70 (pZap70) and Akt (pAkt) in presence of pp65, PBMCs of CMV(+) HIV-uninfected healthy donors were cultured in the presence or absence of pp65 peptide pool (1 µg/ml) for 15, 30, 60 min and 4, 24 and 48 hrs. Cells were surface stained with anti- CD3,-CD4 Abs followed by intracellular staining with anti-pZap70$^{(pY292)}$ or –pAkt$^{(pS473)}$ or isotype control Ab. pZap70 and pAkt in control, and pp65 treated cells were determined in $CD3^{+}CD4^{+}$ cells by flow cytometry. Percent Net $CD3^{+}CD4^{+}$phospho^{+} cells were calculated as: $CD3^{+}CD4^{+}$phospho^{+} cells in control - $CD3^{+}CD4^{+}$phospho^{+} cells in pp65 treated. **(A)** Representative histogram flow cytometry plot is shown for pZap70 *(Upper panel)* and pAkt *(Lower panel)*. **(B)** Mean +/- SD are shown for 3 donors.

Supplemental Figure S3

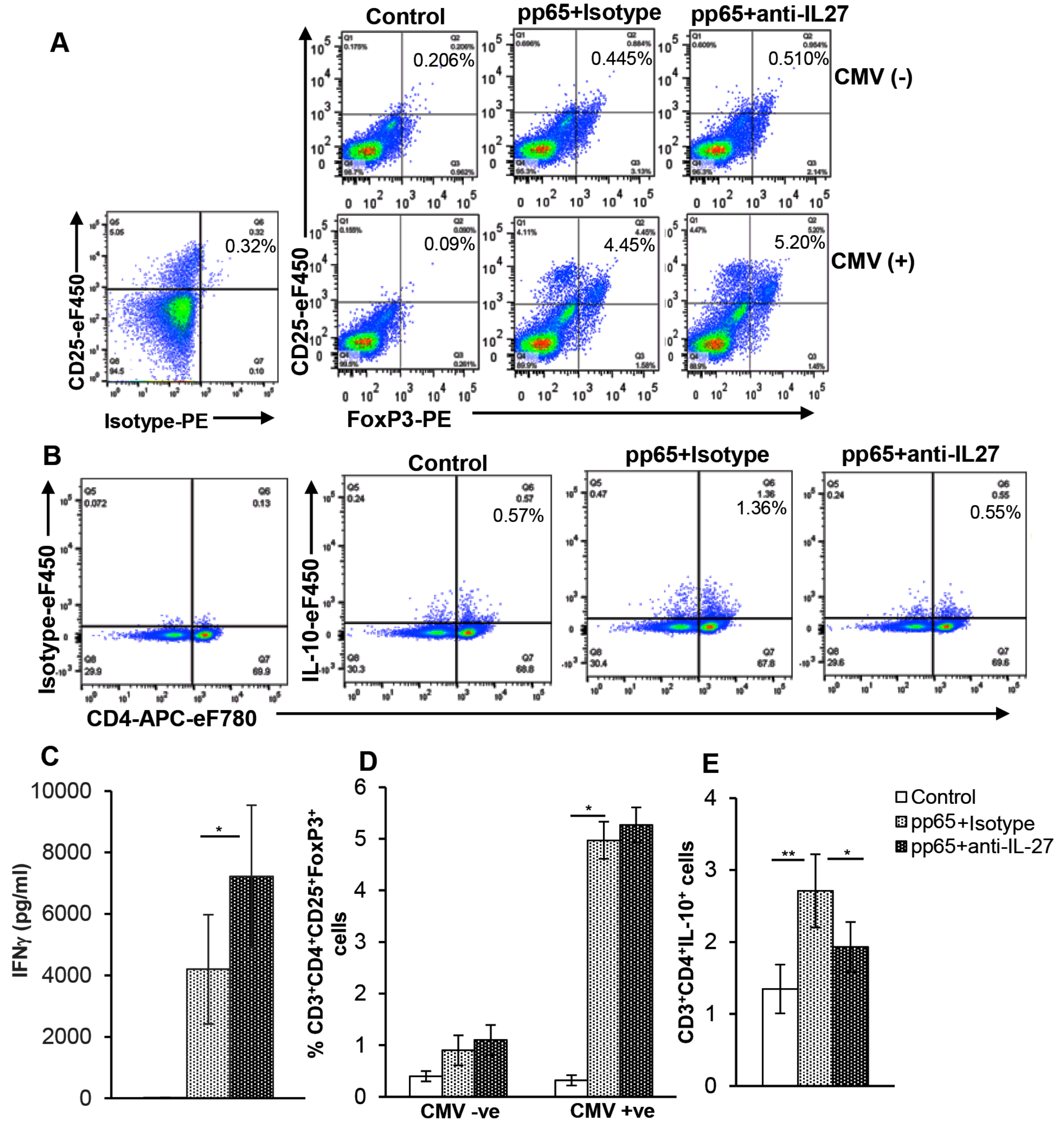

Effect of IL-27 on T cell responses. (A and B) Representative flow cytometry plots showing (A) $CD3^+CD4^+CD25^+FoxP3^+$ Tregs in donor groups as indicated; (B) $CD14^-CD3^+CD4^+IL\text{-}10^+$ cells in CMV(+) HIV-uninfected healthy donors. **(C, D and E)** All the donors were HIV(-) and either CMV(-) or CMV(+). (C) To determine the effect of IL-27 on CMV IFNγ production, PBMCs of CMV (+) HIV(-) (n=6) were cultured with or without CMVpp65 in the presence or absence of neutralizing anti-IL-27 Ab. Supernatants were collected after 48 hrs

and the quantity of IFNγ determined by ELISA. (D) Effect of IL-27 on Treg cell expansion was determined. PBMCs (n=4) from CMV (-) or CMV (+) HIV(-) donors were cultured with or without CMVpp65 in the presence or absence of neutralizing anti-IL27 Ab. After 3 days, cells were surface stained using anti-CD3, -CD4, -CD25 Abs followed by intracellular staining using anti-FoxP3 or isotype control Ab. % $CD3^+CD4^+CD25^+FoxP3^+$ cells were determined by flow cytometry. **(E)** Effect of IL-27 on IL-10 production by $CD4^+$ T cells was determined. PBMCs of CMV (+) HIV(-) (n=6) were cultured with or without the CMVpp65 in the presence or absence of neutralizing anti-IL-27 Ab for 48 hrs. Brefeldin A was added for the last 5 hrs of culture. Cells were surface stained with anti-CD14, -CD3, -CD4, fixed, permeabilized and stained with anti-IL-10 for intracellular IL-10. %$CD14^-CD3^+CD4^+IL\text{-}10^+$ cells was determined by flow cytometry. Mean values +/- SD are shown; *$p<0.05$, **$p<0.005$

Supplemental Table SI

S No	$CD4^+$ T cell count (mm^3)	Viral Load
P01	192	348
P02	383	52623
P03	10	15353
P04	524	5953
P05	16	45066
P06	703	20
P07	465	115
P10	349	21
P11	617	9863
P12	832	33
P13	571	12468
P14	739	20
P15	628	41063
P17	727	20
P18	511	20
P20	413	54392

$CD4^+$ T cell count and Viral Load of the HIV(+) donors.

CD4 T cell count and viral load of HIV (+) individuals was determined; IL-27 quantity in the plasma was calculated and correlated with CD4 T cell count and viral load (Figure 6a & 6b).
